# Identification of a mitophagy-related gene signature for predicting overall survival and response to immunotherapy in rectal cancer

**DOI:** 10.1186/s12885-024-13412-1

**Published:** 2025-01-06

**Authors:** Jian Yang, Zhifei Cao, Chengqing Yu, Wenxu Cui, Jian Zhou

**Affiliations:** 1 Department of General Surgery, The First Affiliated Hospital of Soochow University, Suzhou, 215006 China; 2 Department of Pathology, The Second Affiliated Hospital of Soochow University, Suzhou, 215004 China; 3 State Key Laboratory of Radiation Medicine and Protection, Soochow University, Suzhou, 215123 China

**Keywords:** Mitophagy, Rectal cancer, Prognosis, Signature, Immunotherapy

## Abstract

**Background:**

Rectal cancer is a highly heterogeneous gastrointestinal tumor, and the prognosis for patients with treatment-resistant and metastatic rectal cancer remains poor. Mitophagy, a type of selective autophagy that targets mitochondria, plays a role in promoting or inhibiting tumors; however, the importance of mitophagy-related genes (MRGs) in the prognosis and treatment of rectal cancer is unclear.

**Methods:**

In this study, we used the differentially expressed genes (DEGs) and MRGs from the TCGA-READ dataset to identify differentially expressed mitophagy-related genes (MRDEGs). The mitophagy scores were then analyzed for differential expression and ROC. Seven module genes were identified using the weighted gene coexpression network analysis (WGCNA) approach and subsequently validated in the merged datasets GSE87211 and GSE90627. The model genes were obtained based on prognostic features, and the subgroups were distinguished by risk score. Gene enrichment, immune infiltration and immunotherapy response were also evaluated. Finally, validation of prognostic gene expression in rectal cancer was carried out using clinical samples, employing Immunohistochemistry (IHC).

**Results:**

We demonstrated that 22 MRGs were differentially expressed between normal and rectal cancer tissues. A prognostic model for rectal cancer MRGs was constructed using WGCNA and Cox regression, which exhibited good diagnostic performance. In this study, we identified four molecular markers (*MYLK*,* FLNC*,* MYH11*, and *NEXN*) as potential prognostic biomarkers for rectal cancer for the first time. Moreover, our findings indicate that the risk scores derived from the four MRGs are associated with tumor immunity. To further validate our findings, IHC analyses suggested that the expression of *MYH1*1 in rectal cancer tissues was lower than in nontumorous rectal tissues.

**Conclusion:**

MRGs could predict the prognosis and response to immunotherapy in patients with rectal cancer and might be able to personalize treatment for patients.

**Supplementary Information:**

The online version contains supplementary material available at 10.1186/s12885-024-13412-1.

## Introduction

Colorectal cancer (CRC) is one of the most malignant tumors of the digestive tract and has a poor prognosis. According to GLOBOCAN, in 2020, there were approximately 1.93 million new cases of CRC and 935,000 associated deaths worldwide. The incidence of rectal cancer is estimated at 730,000 cases, with approximately 340,000 deaths [1]. Surgery is a crucial treatment for CRC, yet the specific location of the rectal cancer lesion might result in adverse effects on fertility, sexual function, and bladder function. Additionally, some patients might require permanent colostomy [2]. Therefore, rectal cancer is a primary focus of our research. Despite clinical advancements in combination therapy for rectal cancer, the prognosis for patients with treatment-resistant and metastatic disease remains poor [3, 4]. It is therefore crucial to identify meaningful biomarkers that can facilitate accurate prognostic assessment and individualized treatment.

Autophagy is a self-protective mechanism in eukaryotes that is often activated in cells to counteract various stresses, including starvation, inflammation, injury, and tumors, to maintain homeostatic balance within the organism [5]. Mitophagy is a selective form of autophagy that targets mitochondria. It plays an important role in maintaining mitochondrial homeostasis by selectively removing damaged, folded, and excess mitochondria. The most prevalent neurodegenerative conditions linked to aberrant mitophagy are Alzheimer’s disease (AD) and Parkinson’s disease (PD) [6, 7]. Furthermore, abnormal mitophagy is closely related to tumor development and progression [8, 9]. Mitophagy can act as a tumor-suppressive mechanism by removing damaged mitochondria, though it can also contribute to tumor survival and therapeutic resistance. However, the precise mechanism by which this occurs has yet to be elucidated. To our knowledge, no studies have explored the prognostic and immunotherapeutic potential of mitophagy as a biomarker for rectal cancer.

Preoperative radiotherapy has been demonstrated to reduce the severity of rectal cancer. Nevertheless, resistance to radiotherapy might result in local treatment failure, tumor recurrence, or even metastasis. The results of the previous study indicated that increased basal levels of mitophagy combined with X-ray irradiation reduce G2/M phase arrest, significantly increase DNA damage and promote tumor cell death [10]. It has been demonstrated that radiation therapy can induce mitochondrial damage and disrupt mitochondrial function. Moderate mitophagy has been shown to promote cellular homeostasis, whereas excessive autophagy has been shown to accelerate the induction of cell death [11]. Therefore, identifying mitophagy markers and revealing the underlying mechanisms of clinical therapeutic dilemmas in rectal cancer are highly important.

**Fig. 1:**
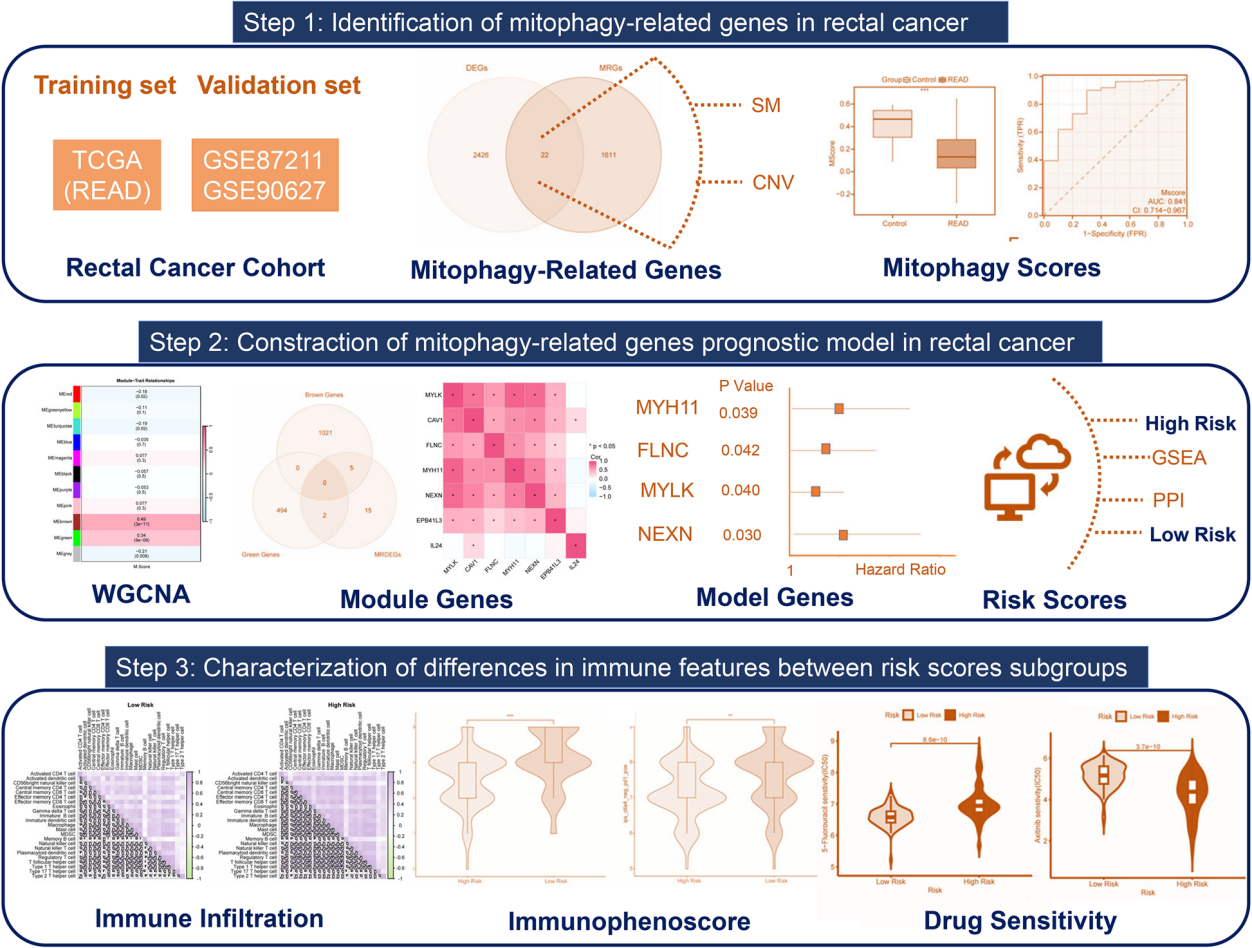
Graphical abstract for the comprehensive characterization of MRGs in rectal cancer

In our study, we attempted to identify differentially expressed mitophagy-related genes (MRDEGs) and and establish the prognostic signature in TCGA-READ dataset. We also investigated the correlation between the prognostic model and gene enrichment analysis, immune infiltration, and immunotherapy responses. The workflow for this study is shown in Fig. 1. The results of this study might contribute to the prediction of patient prognosis and the development of immunotherapies for patients with rectal cancer.

## Materials and methods

### Datasets

RNA-sequencing (FPKM) data, mutation expression data and the clinical information of 160 rectal cancer samples in the TCGA-READ cohort were obtained from the UCSC Xena database [12] (https://xena.ucsc.edu/). The TCGA-READ cohort was used as the training cohort. The GSE90627 [13] and GSE87211 [14] were obtained from the GEO database (https://www.ncbi.nlm.nih.gov/geo/) for further validation. After removing the batch effects of GSE90627 and GSE87211, the combined datasets contained 235 READs and 256 normal controls. The specific information of the samples used in this study is shown in Supplementary Tables 1 and 2.

The GeneCards database [15] (https://www.genecards.org/) was used to collect mitophagy-related genes (MRGs). After the term “mitophagy” was used as a search keyword and only MRGs with “protein coding” and “relevance score > 1” were retained, a total of 1633 MRGs were obtained.

### Construction of the mitophagy score and weighted gene association network analysis (WGCNA)

The R package “DESeq2” was used to perform differential analysis of genes in the READ and normal control. Threshold values of | logFC | > 2.5 and adj. *P* < 0.05 were set for the differentially expressed genes (DEGs). The results of the difference analysis were plotted in volcano plots using the R package “ggplot2”. MRDEGs are the result of the intersection of MRGs and DEGs.

The R package “GSVA” was used to calculate the mitophagy score (M Score) based on the expression matrix of MRDEGs and the TCGA-READ dataset through the ssGSEA. The R package “pROC” was used to draw the ROC curves and calculate the areas under the ROC curve (AUCs). WGCNA [16] was performed using the R package “WGCNA”. The genes (| r | > 0.30) associated with MRDEGs were intersected, and these genes were named module genes.

### Construction and validation of the MRG prognostic signature

Univariate and multivariate Cox regression analyses were performed by R package “survival”. Variables with *p* values < 0.10 in univariate Cox regression were screened by multivariate Cox regression analysis to obtain the model genes of the prognostic risk model. The risk score was calculated using the following formula:

$$\:\text{r}\text{i}\text{s}\text{k}Score\:=\:\sum\limits_{i}Coefficient\:\left({gene}_{i}\right)*mRNA\:Expression\:\left({gene}_{i}\right)$$

The R package “timeROC” was used to draw time-dependent ROC curves and calculate the AUCs based on risk score and overall survival (OS). The R package “rms” was used to construct a nomogram [17] based on the results of multivariate Cox regression analysis. A calibration curve was drawn to evaluate the accuracy and discrimination of the prognostic risk model.

### Molecular characteristics of the risk score subgroup

The TCGA-READ samples were divided into high- and low-risk groups according to the median value of the risk score, and the R package “DESeq2” was used for differential analysis. The thresholds | logFC | > 2.5 and adj. *p* < 0.05 were set for DEGs in the risk group. Based on these DEGs, gene set enrichment analysis (GSEA) was performed using the R package “clusterProfiler”. *P* < 0.05 and an FDR value (q value) < 0.25 were considered statistically significant, and the *p* value correction method was Benjamini‒Hochberg (BH).

STRING [18] was used to construct protein‒protein interaction network (PPI network) with interaction scores > 0.4. Five algorithms in Cytoscape [19] was used: Maximal Clique Centrality (MCC), Maximum Neighborhood Component (MNC), Degree, Edge Percolated Component (EPC), Closeness. Hub Genes were obtained using five different algorithms.

### Analysis of Immune characteristics and drug sensitivity

The R package “gsva” was utilized to conduct ssGSEA [20] to calculate the scores of infiltrating immune cells and to evaluate the activity of immune-related pathways. The immunophenoscores (IPS) data of READ samples were downloaded from the Cancer Immunome Atlas (TCIA) database [21] (https://tCIa.at/home), and the R package “ggplot2” was used to draw a group comparison of the IPS data between high- and low-risk patients. The R package “oncoPredict” and The Genomics of Drug Sensitivity in Cancer (GDSC, https://www.cancerrxgene.org/) were evaluated the half- maximal inhibitory concentration (IC50) of common clinical chemotherapeutic and targeted drugs.

### Clinical specimens and ethics statement

Fifty RC tissues and 24 adjacent nontumor tissues were collected from the First Affiliated Hospital of Soochow University from October 2018 to January 2023. The study protocol was approved by the First Affiliated Hospital of Soochow University Research Ethics Committee (No. 2024215). All experiments were performed in compliance with the relevant regulations, and all patients provided written informed consent.

### Immunohistochemistry

Immunohistochemistry (IHC) was performed on 50 RC tissues and 24 nontumor tissues, which were assessed using an anti-MYH11 antibody (Proteintech. Cat No: 18569-1-AP) according to a standard protocol. Finally, protein expression was assessed by microscopy.

$$\mathrm H-\mathrm{score}\;=\;\mathrm\Sigma\;(\mathrm{pi}\;\times\;\mathrm i)\;=\;(\mathrm{percentage}\;\mathrm{of}\;\mathrm{cells}\;\mathrm{with}\;\mathrm{weak}\;\mathrm{staining}\;\mathrm{intensity}\;\times\;1)\;+\;(\mathrm{percentage}\;\mathrm{of}\;\mathrm{cells}\;\mathrm{with}\;\mathrm{medium}\;\mathrm{staining}\;\mathrm{intensity}\;\times\;2)\;+\;(\mathrm{percentage}\;\mathrm{of}\;\mathrm{cells}\;\mathrm{with}\;\mathrm{strong}\;\mathrm{staining}\;\mathrm{intensity}\;\times\;3)$$

### Statistical analysis

All data processing and analysis in this study were performed with R software (version 4.2.2). The Wilcoxon rank sum test (Mann–Whitney U test) compared two groups, while the Kruskal–Wallis test assessed three or more groups. Spearman correlation analysis calculated correlation coefficients between different molecules, with a significance thresh- old at *P* < 0.05.

## Results

### Coalescence of rectal cancer datasets

R package “sva” was used to remove batch effects in GSE90627 and GSE87211 to obtain the combined GEO datasets. The distribution boxplot (Supplementary Fig. 1A-B) was subsequently used to compare the expression values of the datasets before and after removing the batch effects. Principal component analysis (PCA) plot (Supplementary Fig. 1C-D) was used to compare the distributions of low-dimensional features before and after batch effect removal. These results showed that the batch effects of samples in the READ dataset were essentially eliminated after batch removal.

### Differentially expressed genes related to mitophagy in rectal cancer

**Fig. 2:**
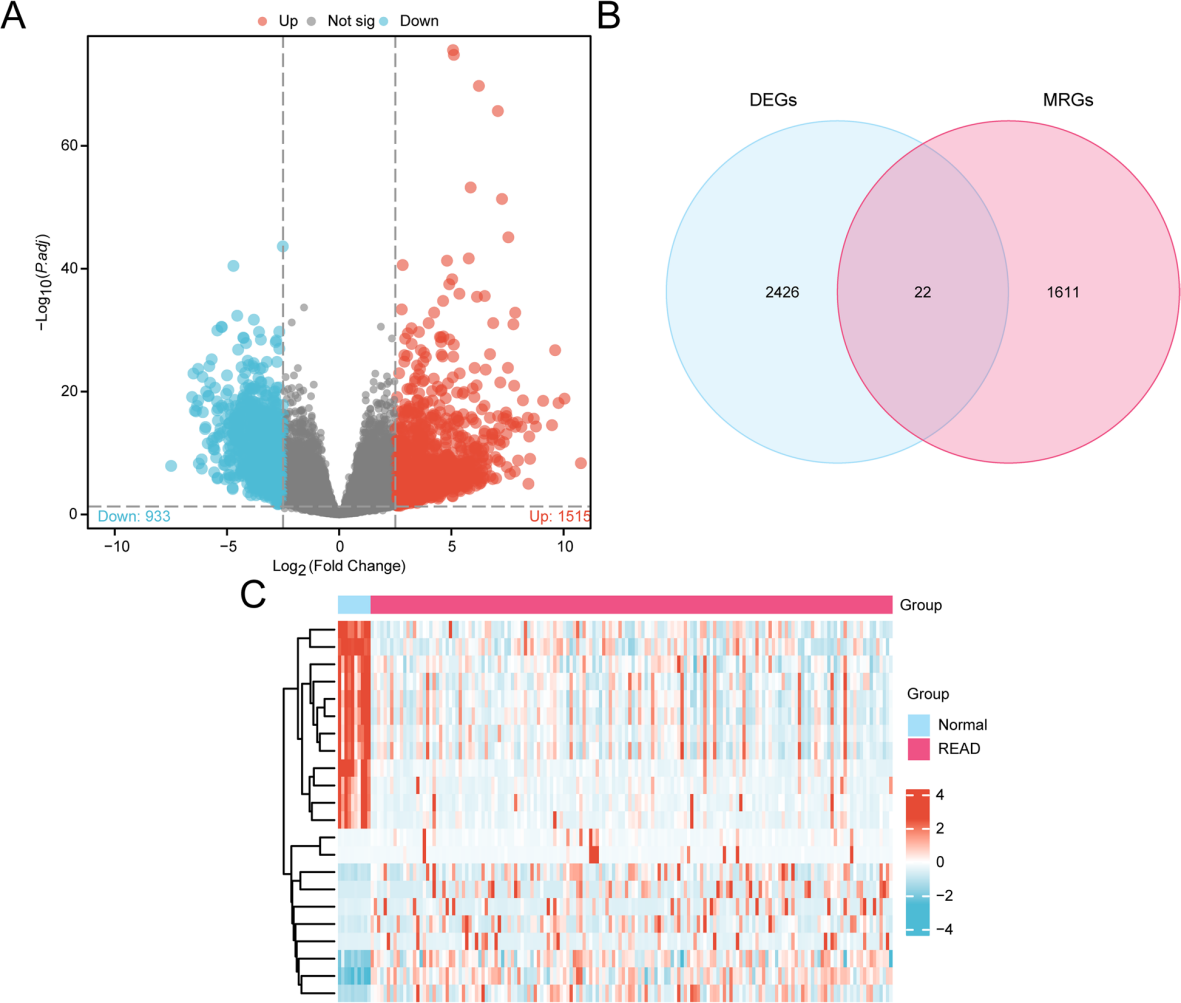
Differential gene expression analysis. **A** Volcano plot of differentially expressed genes between READ samples and normal controls in the TCGA-READ cohort. **B** Venn diagram of DEGs and MRGs in the TCGA-READ cohort. **C** Heatmap of MRDEGs in the TCGA-READ cohort

A total of 2448 DEGs were identified, of which 1515 were up-regulated genes and 933 were down-regulated genes (Fig. 2A). To obtain the MRDEGs, the intersection of all the DEGs and the MRGs obtained was taken and plotted as a Venn diagram (Fig. 2B). A total of 22 MRDEGs were obtained: *SPTBN2*,* MYLK*,* CA2*,* NEFM*,* CAV1*,* MYH11*,* MAPK15*,* LRCH2*,* FLNC*,* PSAT1*,* EPB41L3*,* TUBB4A*,* IGF2BP1*,* CDKN2A*,* KIF1A*,* NEXN*,* DPYSL5*,* MIF*,* IL24*,* AHSG*,* IGF2BP3* and *ALB*. The heatmap results showed that the expression of MRDEGs differed between READ and normal control samples. (Fig. 2C).

### Analysis of somatic mutation (SM) and copy number variation (CNV) of MRDEGs

**Fig. 3:**
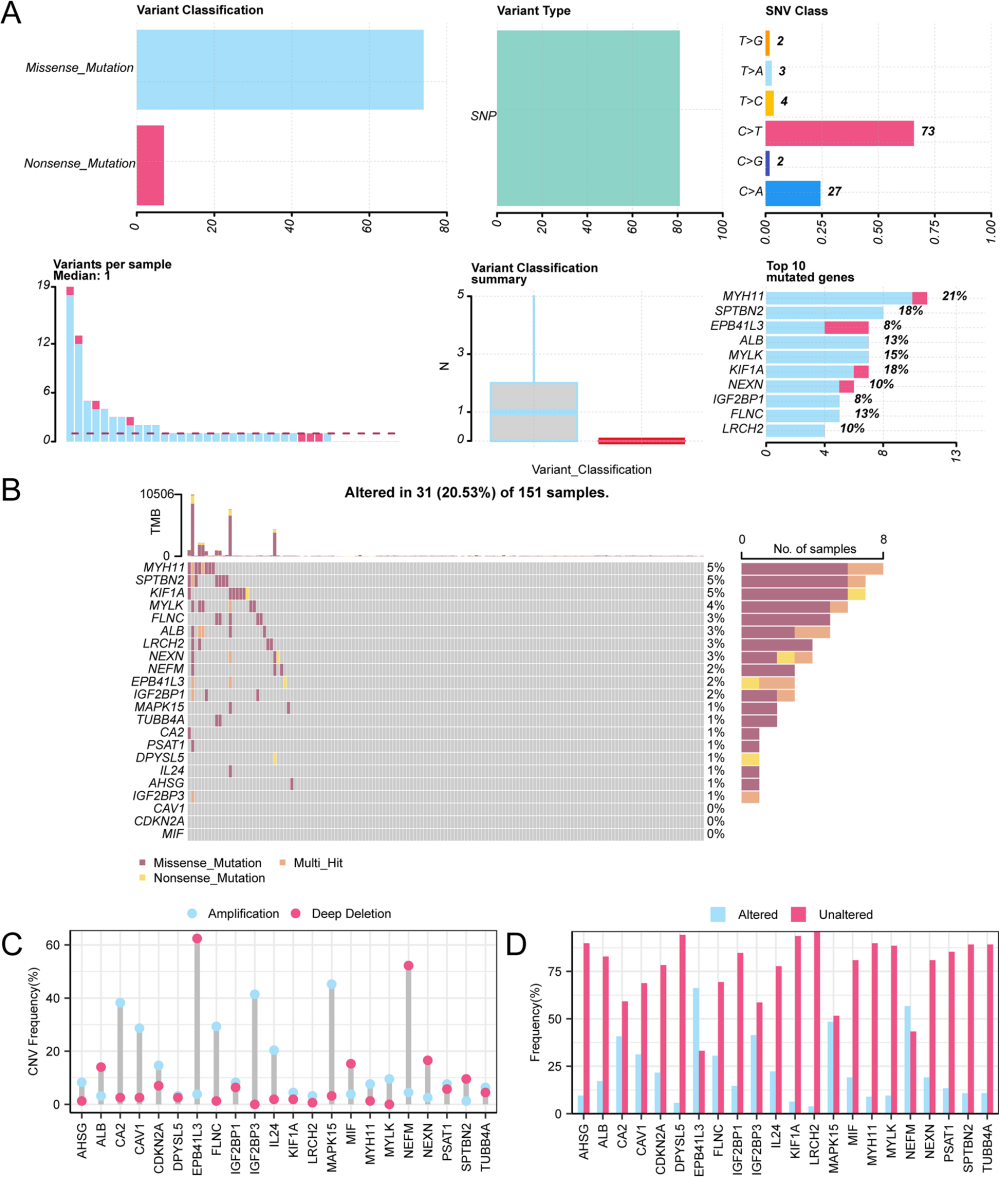
Somatic mutation and CNV analysis. **A**, **B** Display of somatic mutations (SMs) in the MRDEGs from the READ cohort. **C**, **D** MRDEGs with copy number variations (CNVs) are shown in the READ cohort

We analyzed the mutations of 22 MRDEGs in READ (Fig. 3A). The results revealed two main types of somatic mutations (SMs) in MRDEGs, and missense mutations accounted for the majority of the mutations. In addition, the mutation types of the 22 MRDEGs in READ mainly consisted of single-nucleotide polymorphisms (SNPs), and C-to-T mutations were the most common single-nucleotide variants (SNVs). 22 MRDEGs were sorted by mutation frequency from high to low, and the results revealed that *MYH11* had the highest mutation rate, with a mutation rate of 5% (Fig. 3B). Through GISTIC2.0 analysis, a total of 22 MRDEGs were found to have copy number variations (CNVs) in READ, and the mutation statuses of the 22 genes with CNVs are shown (Fig. 3C-D).

### Construction of the mitophagy score and WGCNA

**Fig. 4:**
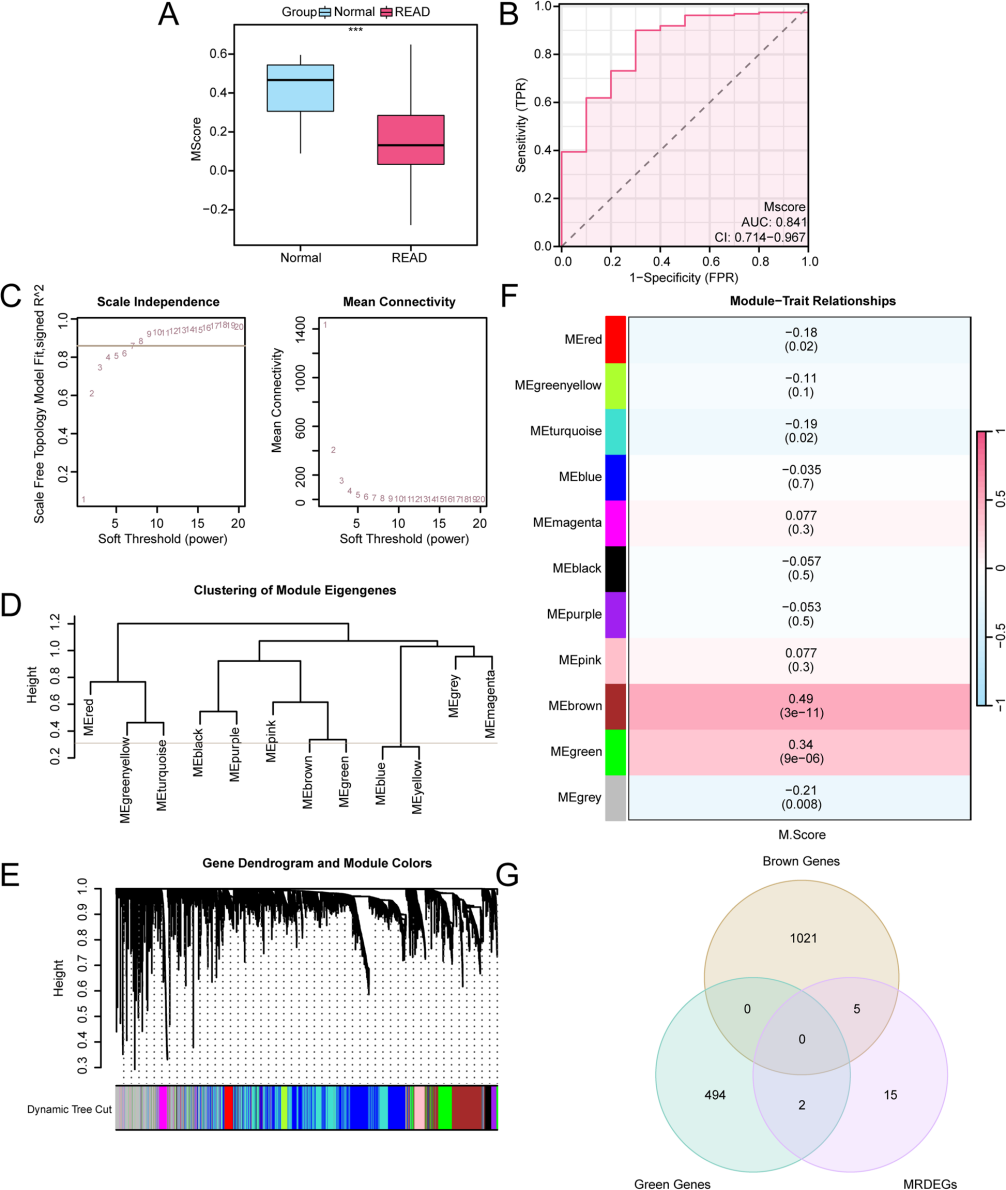
WGCNA for TCGA-READ. **A** Group comparison plot results of the M Scores between READ and normal controls. **B** ROC curve of the M Scores in the TCGA-READ cohort. **C** Scale-free network display of the best soft threshold from WGCNA; the left panel shows the best soft threshold, and the right panel shows the network connectivity under different soft thresholds. **D** Display of module clustering results of genes with the top 30% variance. **E** Presentation of cluster results for genes. The upper part is divided into a hierarchical clustering dendrogram, and the lower part is divided into gene modules. **F** Results of the correlation analysis between the gene cluster modules and the M Score. **G** Venn diagram of 22 MRDEGs and MEbrown and MEgreen module genes

Based on the expression of the 22 MRDEGs in the TCGA-READ cohort, the M Score values of all the samples were calculated using the ssGSEA algorithm. Figure 4A shows that the M Score expression was highly significantly different (*p* value < 0.001) between READ patients and normal controls. In addition, the ROC curve (Fig. 4B) revealed that M Score values showed a certain degree of accuracy (AUC = 0.841).

WGCNA was performed on genes in the top 30% of variance in READ to screen for coexpression modules. The scale-free fit indices (Fig. 4C) under different soft thresholds were calculated, and genes were clustered in a clustering tree (Fig. 4D). The results revealed that when the screening criterion was 0.3, the genes clustered into 11 modules (Fig. 4E). With |r value| > 0.30 as a criterion, MEbrown (|r value| = 0.49) and MEgreen (|r value| = 0.34), were selected for further analysis (Fig. 4F). A total of 7 module genes have been obtained from the 22 MRDEGs and from these two screening modules (Fig. 4G), namely, *MYLK*,* CAV1*,* FLNC*,* MYH11*,* NEXN*,* EPB41L3* and *IL24*.

### Module gene expression differences and correlation analysis

**Fig. 5:**
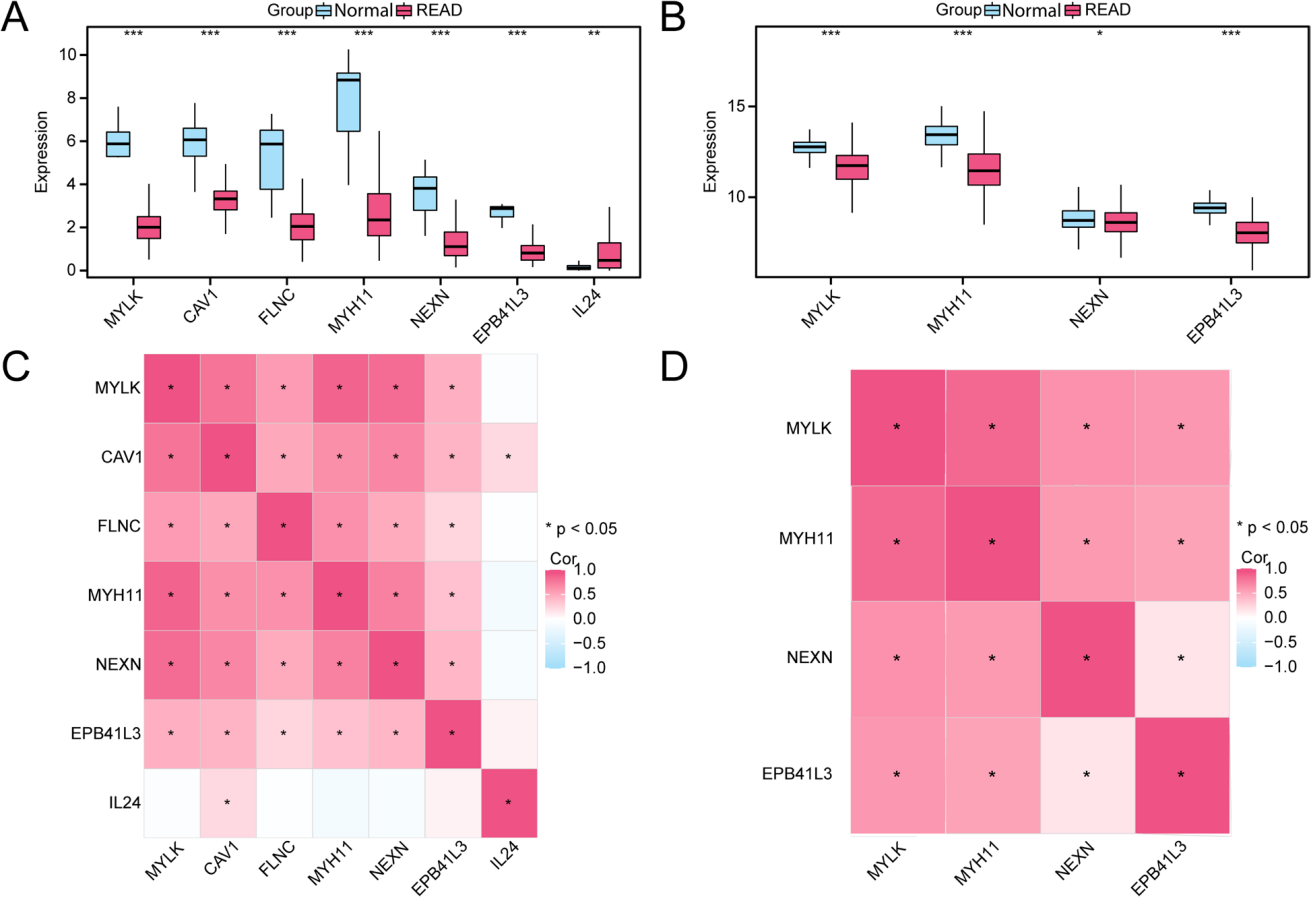
Differential expression and correlation analysis. **A**, **B** Group comparison diagram of module genes in the READ and normal controls in the TCGA-READ dataset (**A**) and in the combined GEO dataset (**B**). **C**, **D** Heatmap of correlations between module genes in the TCGA-READ (**C**) and combined GEO datasets (**D**)

The seven module genes whose expression levels were statistically significant (*p* < 0.05) in READ patients and normal controls in the TCGA-READ cohort (Fig. 5A). A total of 4 module genes were significantly (*p* < 0.05) expressed in two groups of the integrated GEO dataset: *MYLK*,* MYH11*,* NEXN*, and *EPB41L3* (Fig. 5B). The results of pairwise correlations revealed that there were mainly positive correlations between module genes in the TCGA-READ dataset and in the combined GEO datasets (Fig. 5C-D).

### Construction and validation of the MRG prognostic signature

To construct a prognostic risk model for READ, the univariate and multivariate Cox regression were used to explore the correlation between risk score expression and clinical prognosis (Supplementary Fig. 2A-B). Four module genes were screened: *MYLK*,* FLNC*,* MYH11* and *NEXN*, and named model genes. The risk score was calculated using the following formula:

$$\:\text{R}\text{i}\text{s}\text{k}\:\text{S}\text{c}\text{o}\text{r}\text{e}\:=\:\text{M}\text{Y}\text{L}\text{K}\:\text{*}\:\left(0.0301\right)\:+\:\text{F}\text{L}\text{N}\text{C}\:\text{*}\:\left(0.125\right)\:+\:\text{M}\text{Y}\text{H}11\text{*}\:\left(0.031\right)\:+\:\text{N}\text{E}\text{X}\text{N}\:\text{*}\:\left(0.225\right)$$

**Fig. 6:**
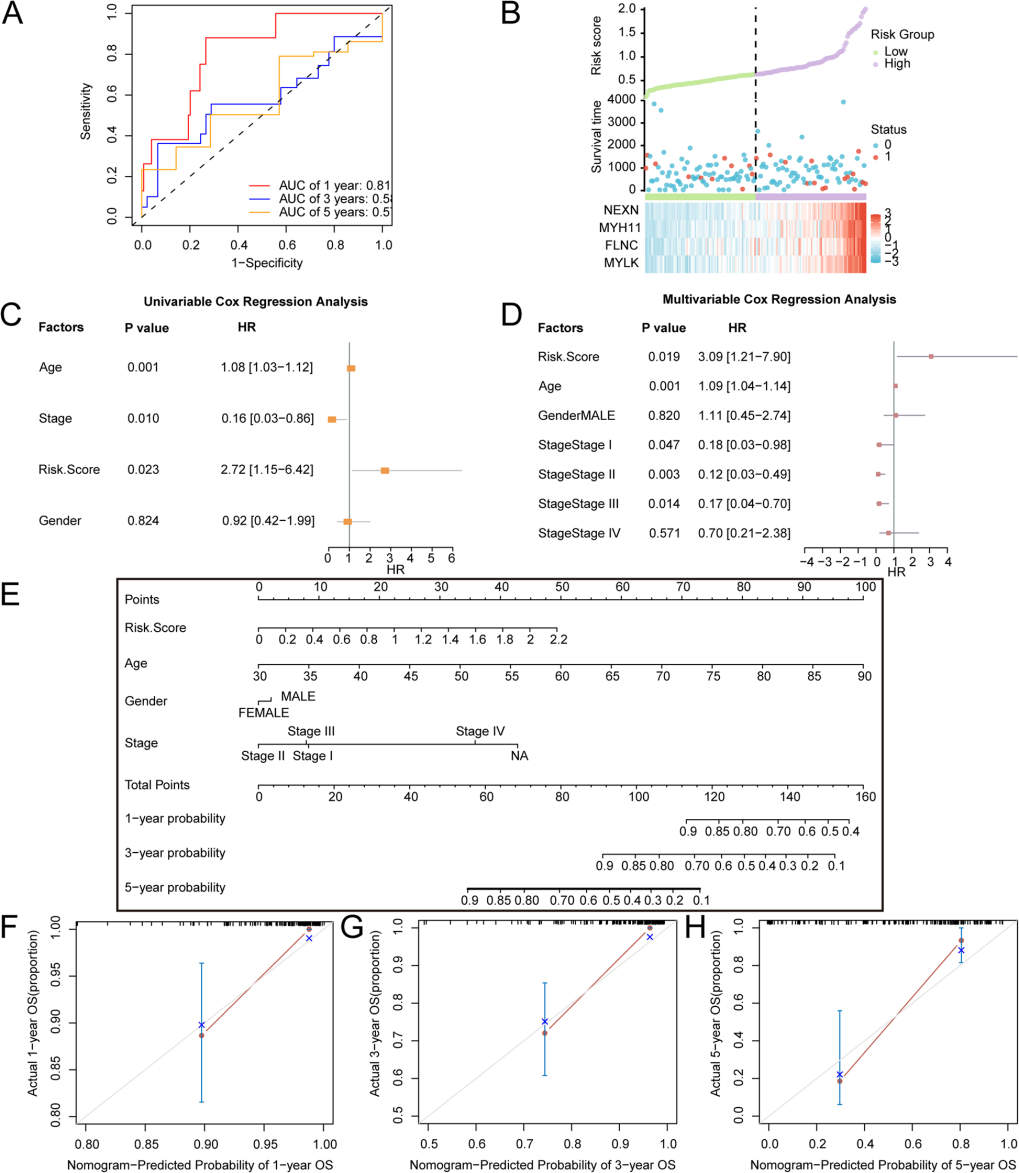
Prognostic analysis. **A** Time‒dependent ROC curves of the TCGA cohort. **B** Risk factor plot of the TCGA cohort. **C **Univariate Cox regression. **D** Multivariate Cox regression. **E** Nomogram of the risk score and clinical information. **F**-**H** Calibration curves of 1-year (**F**), 3-year (**G**), and 5-year (**H**)

The results of the time ROC curve (Fig. 6A) indicated that the prognostic risk model had some accuracy at year 1 (0.7 < AUC < 0.9). The results of risk factor map revealed that prognostic risk model genes were highly expressed in the high-risk group (Fig. 6B). The results of Cox regression analyses revealed that risk score, clinical stage and age were statistically significant (Fig. 6C-D, Supplementary Table 3). A nomogram was drawn based on risk score and clinical information, and the results revealed that the risk score and stage played important roles (Fig. 6E). The results of the calibration curve analysis revealed that the prognostic risk model of READ had the best clinical prediction effect (Fig. 6F-H).

### Enrichment analysis of the DEGs of the risk score subgroup

**Fig. 7:**
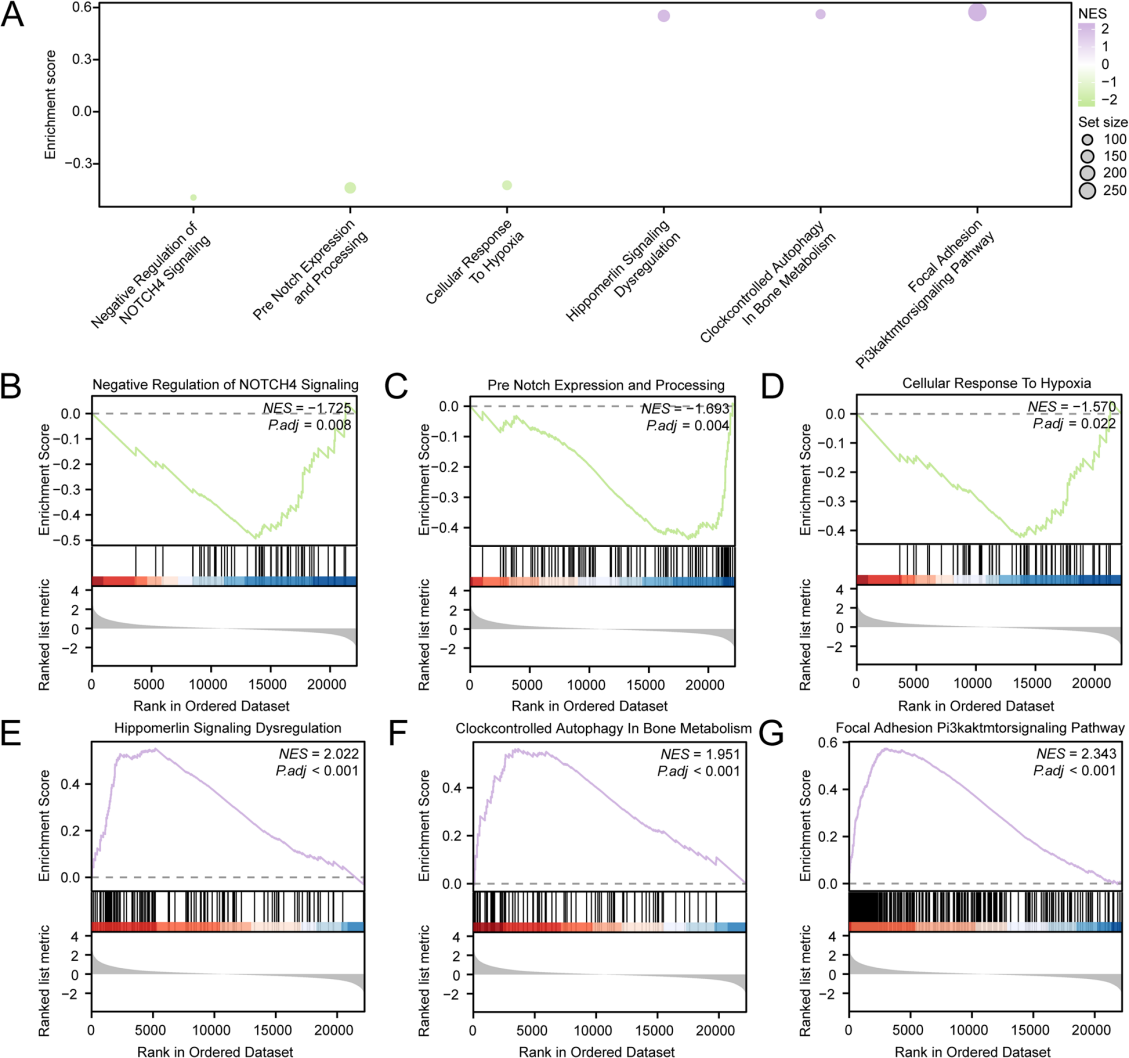
GSEA for the TCGA-READ Risk Group. **A** Bubble plot of GSEA results. **B**-**G** Genes were significantly enriched in the Negative Regulation of NOTCH4 Signaling (**B**), Pre-NOTCH Expression and Processing (**C**), Cellular Response to Hypoxia (**D**), Hippo merlin Signaling Dysregulation (**E**), Clock Controlled Autophagy in Bone Metabolism (**F**), Focal Adhesion PI3K/Akt/mTOR Signaling Pathway (**G**)

READ samples in TCGA were divided into high- and low-risk groups according to the median risk score. There were 70 DEGs between the two subgroups, of which 66 genes were upregulated and 4 genes were downregulated (Supplementary Fig. 3. A-B). GSEA was used to determine the effects of the expression levels of all genes in the high- and low-risk groups on their molecular functions (Fig. 7A, Supplementary Table 4). The results revealed that the genes were significantly enriched in the Negative Regulation of NOTCH4 Signaling (Fig. 7B), Pre-NOTCH Expression and Processing (Fig. 7C), Cellular Response to Hypoxia (Fig. 7D), Hippo merlin Signaling Dysregulation (Fig. 7E), Clock Controlled Autophagy in Bone Metabolism (Fig. 7F), Focal Adhesion PI3K/Akt/mTOR Signaling Pathway (Fig. 7G).

Using STRING database, a PPI network of 70 DEGs was constructed, among which 31 proteins were highly associated (Supplementary Fig. 4A). Five algorithms were applied in Cytoscape software to map the top 10 proteins, respectively (Supplementary Fig. 4B-F). The 7 Hub Genes were obtained by intersecting 5 algorithms, which are as follows: *MYH11*,* SYNPO2*,* CNN1*,* PLN*,* DES*,* CASQ2* and *SYNM* (Supplementary Fig. 4G).

### Immune characteristics and drug sensitivity of the risk score subgroups

**Fig. 8:**
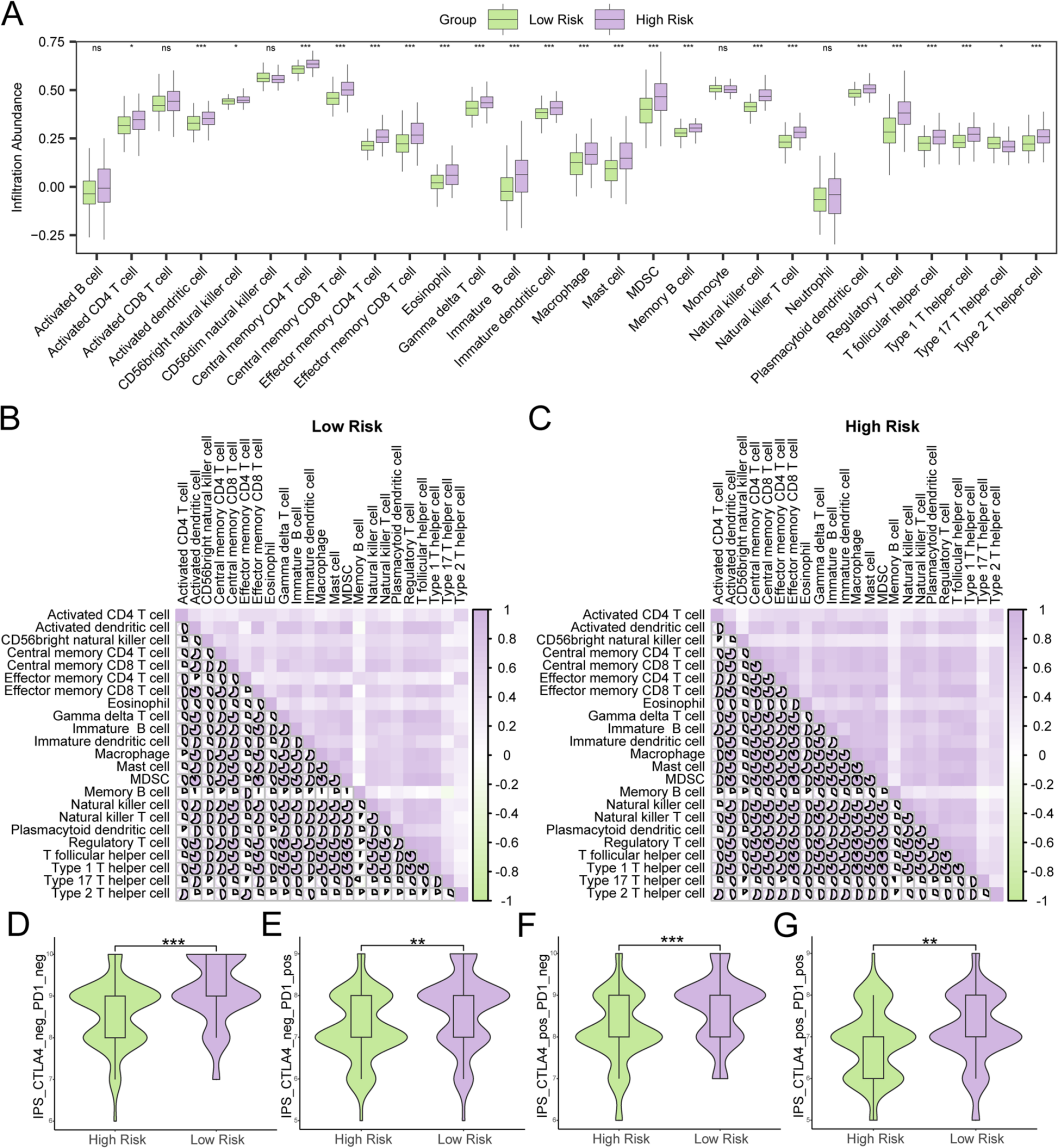
Risk group immune infiltration analysis by ssGSEA. **A** Comparison of the grouping of immune cells in the low- and high-risk groups of the READ samples. **B**, **C** Correlation analysis of immune cell infiltration in the low-risk group (**B**) and high-risk group (**C**). **D**-**G** Comparison of different IPSs in the high- and low-risk groups in the TCGA-READ cohort: ips_ctla4_neg_pd1_neg (**D**), ips_ctla4_neg_pd1_pos (**E**), ips_ctla4_pos_pd1_neg (**F**), and ips_ctla4_pos_pd1_pos (**G**)

The expression matrix of the TCGA-READ cohort was used to calculate the immune infiltration abundances of 28 immune cell by ssGSEA. The results revealed that 23 immune cell types were statistically significant (*p* < 0.05), including activated CD4 T cells, activated dendritic cells, central memory CD4 T cells, central memory CD8 T cells, effector memory CD4 T cells, effector memory CD8 T cells, gamma delta T cells, macrophages, memory B cells, natural killer T cells, type 1 T helper cells, type 17 T helper cells, and type 2 T helper cells, etc. (Fig. 8A). Furthermore, most immune cells in the low-risk group of the READ sample were strongly positively correlated, similar to those in the high-risk group (Fig. 8B-C). To analyze the prediction of immunotherapy efficacy in the high- and low-risk groups, we downloaded the IPS associated with the READ samples from the TCIA database and investigated the associations between immune checkpoint inhibitors (ICIs) and risk score subgroups. The IPS between the high- and low-risk groups revealed that patients at low risk had a greater IPS for anti-PD-1 and anti-CTLA4 immunotherapy, which suggested a better immunotherapy response (*p* < 0.001, *p* < 0.01) (Fig. 8D-G).

**Fig. 9:**
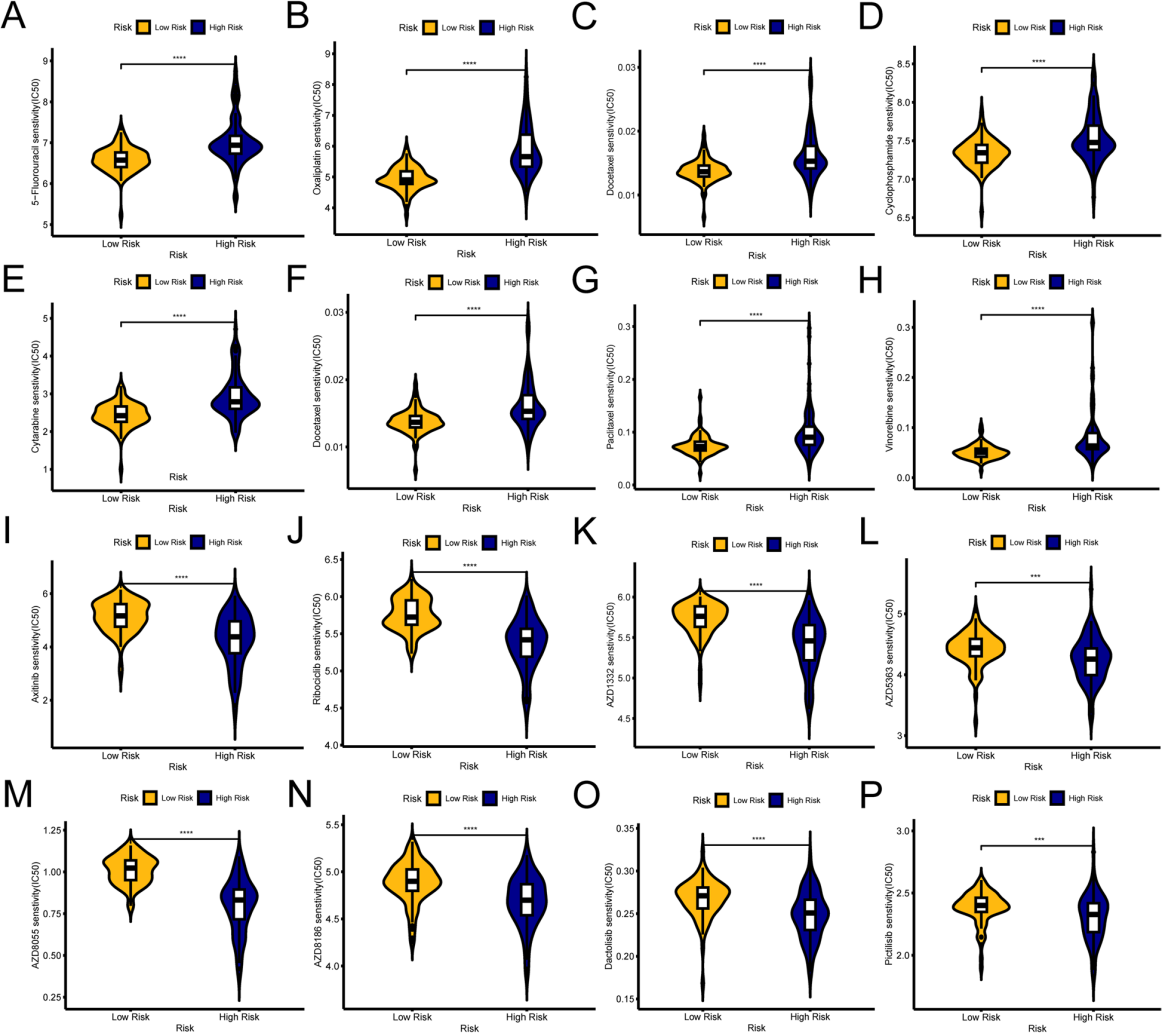
IPS and drug sensitivity analysis. **A**-**P** The IC50 values of 16 chemotherapeutic and targeted drugs were analyzed and compared between the low- and high-risk groups

Chemotherapy and targeted therapies are also commonly used in the treatment of locally advanced RC or those with metastatic disease. We investigated whether there were disparities in responsiveness to chemotherapeutic or targeted agents when stratified by risk score. The IC50 values of clinically utilized chemotherapeutic agents were observed to be lower in the low-risk cohort, indicating that patients at low risk may respond better to most chemotherapy drugs, including 5-Fluorouracil, Oxaliplatin, Docetaxel, Cyclophosphamide, etc. (*p* < 0.0001,) (Fig. 9A-H). In contrast, the high-risk group with an absence of response to immunotherapy also exhibited insensitivity to chemotherapy. However, targeted therapies offer a degree of benefit, including tyrosine kinase inhibitors (Axitinib) (Fig. 9I), CDK4/6 inhibitors (Ribociclib) (Fig. 9J), and RKT pathway inhibitors (AZD1332) (Fig. 9K), PI3K/AKT/mTOR pathway inhibitors (AZD5363, AZD8055, AZD8186, Dactolisib, Pictilisib) (Fig. 9L-P).

### The expression of MYH11 in patients with RC

**Fig. 10:**
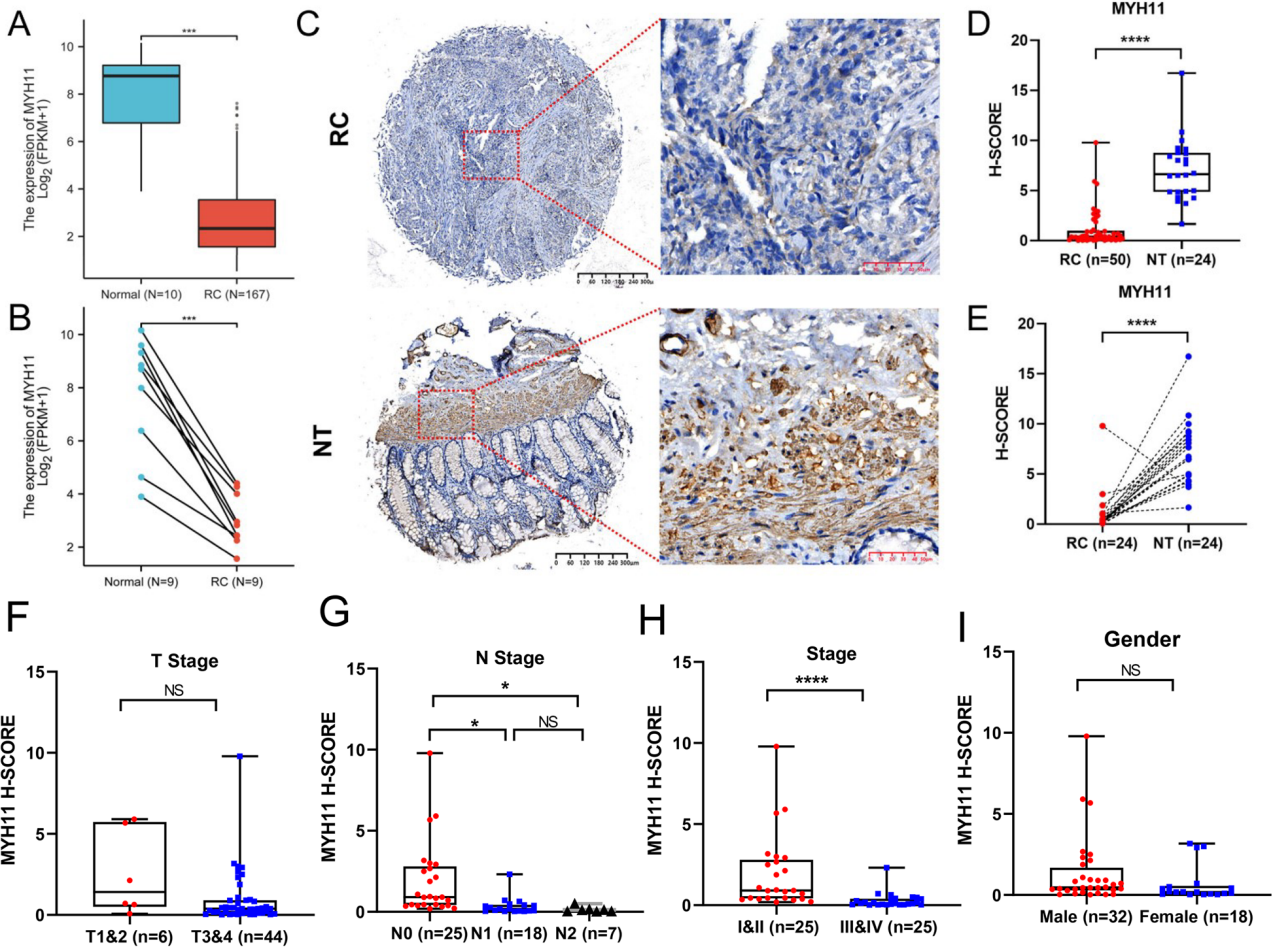
MYH11 expression. **A** Relative expression levels of MYH11 in 167 rectal cancer tissues and 10 normal tissues from the TCGA database. **B** Relative expression levels of MYH11 in 9 rectal cancer tissues and paired adjacent normal tissues from the TCGA database. **C** Representative IHC images of MYH11 in tumors and nontumorous rectal tissues. **D** H score of MYH11 in 50 rectal cancer tissues and 24 nontumorous tissues. **E** H score of MYH11 in 24 rectal cancer tissues and paired nontumorous tissues. **F-I** MYH11 expression and clinicopathological characteristics: T stage (**F**), N stage (**G**), pathologic stage (**H**) and gender (**I**)

Interestingly, MYH11, as one of the four module genes, is also a hub gene. Therefore, we further validated its expression in rectal cancer tissue. In the TCGA cohort, MYH11 was found to be more significantly downregulated in RC tissues than in adjacent normal tissues (*p* < 0.001; Fig. 10A-B). IHC staining for MYH11 in RC and adjacent nontumorous tissue from our hospital was performed. Representative IHC images are shown in Fig. 10C. MYH11 was localized mainly in the cytoplasm of the samples. MYH11 expression was significantly lower in RC tissues than in nontumorous rectal tissues (*p* < 0.0001; Fig. 10D-E). Associations between MYH11 expression and patient clinicopathological characteristics were analyzed, revealing lower levels of MYH11 expression to be evident in RC patients with higher N stage and pathologic stage. In contrast, MYH11 expression was unrelated to T stage or gender (Fig. 10F–I).

## Discussions

Mitophagy is essential for maintaining mitochondrial and cellular homeostasis. To maintain the stability of the mitochondrial network and the intracellular environment, the cell employs autophagy mechanisms to selectively wrap and degrade damaged or dysfunctional mitochondria within the cell [5]. A state of impaired mitophagy could result in abnormal mitochondrial function and, subsequently, a range of chronic diseases. When neuronal mitochondria fail to be completely cleared by mitophagy, the oxidative substances produced can lead to neurodegenerative diseases, such as Alzheimer’s disease (AD), Parkinson’s disease (PD), amyotrophic lateral sclerosis (ALS), and Huntington’s chorea (HD), among others [6, 7]. Additionally, enhancing mitochondrial autophagy could impede the progression in the early stages of heart failure. Conversely, excessive activation of mitophagy is prevalent in the later stages of heart failure, resulting in cardiomyocyte dysfunction and exacerbating heart failure [22, 23].

The precise role of mitophagy in the development of cancer has not been elucidated. A growing body of evidence suggests that mitophagy has the potential to inhibit tumor growth by removing dysfunctional mitochondria. The *PINK1/Parkin* pathway is considered to be the primary pathway of mitophagy. The loss of Parkin function impairs mitophagy, which in turn results in the accumulation of reactive oxygen species (ROS) and thus promotes tumorigenesis [24]. In contrast, hypoxia might also stimulate autophagy in colon cancer cells through the modulation of the translation of the highly conserved lysosomal glycoproteins *PSAP* and *LAMP2*, which in turn increase mitophagy and thus protect tumor cells [25]. Furthermore, mitophagy can be activated via oncogenic signal transduction pathways (principally including the TGFβ and NF-κB pathways) to facilitate tumor cell growth by regulating cancer cell metabolism [26, 27]. Similarly, the relationship between mitophagy and drug resistance in tumor cells remains disputed. Several studies suggest that mitophagy may be a driver of chemoresistance in small cell lung cancer [28], hepatocellular carcinoma [29] and pancreatic cancer [30]. However, mitochondrial autophagy was also shown to overcome chemoresistance in tumor cells. For example, targeting CRL4 can inhibit the growth of chemoresistant ovarian cancer cells by inducing mitophagy [31].

Most studies in the field of biosignature analysis have concentrated on colorectal cancer, whereas only a minority have specifically addressed the issue of rectal cancer. Zhao et al. conducted differential gene screening to identify three hub genes (*PLAGL2*,* ZNF337*, and *ALG10*) in READ radiotherapy responders [32]. Liu et al. constructed prognostic models for rectal and colon cancers using glycolysis-related genes, and the two models were used to construct time-dependent ROCs in rectal cancer with AUCs at 1 year of 0.688 and 0.755, respectively [33]. In contrast, our findings indicated that four model genes presented an AUC value of 0.81 in this study. Our investigation revealed that the combined prognostic performance of these four model genes related to mitochondria exhibited superior predictive ability.

Previous studies have revealed that four specific genes, *MYLK*,* FLNC*,* NEXN* and *MYH11*, are significantly associated with tumors. Myosin light chain kinase (*MYLK*) is a regulatory enzyme that modifies the light chains of myosin, a protein that is crucial for controlling muscle contraction and relaxation [34]. *MYLK* is highly expressed in bladder cancer and is associated with a poor clinical prognosis [35]. However, the expression of *MYLK* in non-small cell lung cancer tissues was significantly lower than those in paracancerous and normal tissues [36], findings that are consistent with our studies. Myosin heavy chain 11 (*MYH11*), encoded by the *MYH11* gene, is a protein that participates in muscle contraction by catalyzing the hydrolysis of adenosine triphosphate (ATP) [37]. A previous study indicated that *MYH11* gene expression is reduced in patients with colorectal cancer or lung cancer, which is correlated with a poor prognosis [38–40]. Furthermore, in gastric cancer, *MYH11* was found to be reduced in GC, whereas *MYH11* upregulation has been shown to inhibit tumor growth [41]. Interestingly, *MYLK*,* MYH11*, and *FLNC* were identified as pivotal genes in studies of the immune microenvironment [42], and *MYLK* and *FLNC* were found to be associated with prostate pathogenesis and prognosis [43].

Patients with CRC with defective mismatch repair (dMMR) or microsatellite high instability (MSI-H) represent the principal beneficiaries of immunotherapy; however, these patients account for only 10–15% of all patients with CRC [44]. A considerably larger proportion of patients with mismatch repair proficient (pMMR) or microsatellite stable (MSS) tumors have “cold tumors” that are not responsive to immunotherapy. A few clinical trials are currently underway to investigate the efficacy of immunotherapy in combination with other therapies in patients with pMMR/MSS CRC. Identifying the dominant subgroups or excluding the nonbeneficial subgroups is highly clinically important. The findings of the abovementioned study indicate that patients at low risk had a greater IPS of anti-PD-1 and anti-CTLA4 immunotherapy, which suggested a better immunotherapy response. Chemotherapy and targeted therapies are also commonly used in the treatment of locally advanced RC or those with metastatic disease. Our results demonstrated that Low-risk patients may experience better chemotherapy outcomes. In contrast, the high-risk group with an absence of response to immunotherapy also exhibited insensitivity to chemotherapy. However, targeted therapies offer a degree of benefit. We believe that the risk score of MRGs could be a useful indicator for guiding the clinical management of RC.

The current study offers new insight into the relationship between MRG expression and prognosis in patients with rectal cancer. However, there are limitations to consider. The present study was based on two publicly available datasets (TCGA and GEO). Most patients in these datasets were from Western countries. As a result, caution should be exercised in applying these results to patients in Asian countries. To validate and improve the clinical utility of the signature, independent studies with larger sample sizes are needed. Second, the spatial heterogeneity of a tumor sample and the lack of multilocus RNA sequence data sampling within individual tumors in public large-scale datasets might result in the model scores being less predictive than they could be. Finally, the biological functions and potential mechanisms of mitophagy in rectal cancer require further studies and rigorous experimental validation in vivo and in vitro.

## Conclusions

In conclusion, the results of our study demonstrated that 22 MRGs were differentially expressed between normal and rectal cancer tissues. A prognostic model for rectal cancer MRGs was constructed using WGCNA and Cox regression, which exhibited good diagnostic performance. In this study, we identified four molecular markers (*MYLK*,* FLNC*,* MYH11*, and *NEXN*) as potential prognostic biomarkers for rectal cancer for the first time. Moreover, our findings indicate that the risk scores derived from these four MRGs are associated with tumor immunity. These observations extend previous research on MRGs in patients with rectal cancer. Based on these results, we intend to conduct biological experiments to further substantiate our conclusions.

## Supplementary Information

Supplementary Material 1: Supplementary Fig. 1. Removal of Batch Effects from GSE90627 and GSE87211. A. Box plot of the combined GEO dataset distribution before batch removal. B. Postbatch integrated GEO dataset (combined dataset) distribution boxplots. C. PCA plot of the datasets before normalization. D. PCA plot of the dataset after normalization.

Supplementary Material 2: Supplementary Fig. 2. Cox Regression Analysis. A. Forest plot of the four model genes in the univariate Cox regression model. B. Forest plot of the four model genes in the multivariate Cox regression model.

Supplementary Material 3: Supplementary Fig. 3. Differential Gene Expression Analysis for Risk Groups. A. Volcano plot of DEGs associated with high- and low-risk patients in the TCGA-READ cohort. B. Heatmap of DEGs in the high- and low-risk groups. TCGA, The Cancer Genome Atlas; READ, rectal cancer; DEGs, differentially expressed genes.

Supplementary Material 4: Supplementary Fig. 4. PPI Network and Hub Genes Analysis. A. PPI network of DEGs in the risk group calculated by the STRING database. B-F. PPI network of the top 10 genes by the 5 algorithms of MCC (B), MNC (C), degree (D), EPC (E) and closeness (F). G. Venn diagram. TCGA, The Cancer Genome Atlas; READ, rectal cancer; PPI network, protein‒protein interaction network; DEGs, differentially expressed genes.

Supplementary Material 5.

Supplementary Material 6.

Supplementary Material 7.

Supplementary Material 8.

## Data Availability

Publicly available data sets were utilized in this study. The details are as follows: TCGA-READ cohort were obtained from the UCSC Xena database (https://xena.ucsc.edu/), the GEO repository (https://www.ncbi.nlm.nih.gov/).Data is provided within the manuscript or supplementary information files.

## References

[1] Hossain MS, Karuniawati H, Jairoun AA, Urbi Z, Ooi J, John A, Lim YC, Kibria KMK, Mohiuddin AKM, Ming LC et al. Colorectal Cancer: a review of Carcinogenesis, Global Epidemiology, Current challenges, risk factors, preventive and treatment strategies. Cancers (Basel) 2022;14(7):1732. 10.3390/cancers14071732 PMC8996939 35406504

[2] Slattery ML, Curtin K, Wolff RK, Boucher KM, Sweeney C, Edwards S, Caan BJ, Samowitz W. A comparison of colon and rectal somatic DNA alterations. Dis Colon Rectum. 2009;52(7):1304–11. 19571709 10.1007/DCR.0b013e3181a0e5df PMC2718791

[3] Yan Z, Yuan Q, He Y, Peng F, Liu Y, Zhang H, Ji X, He X, Zhao Q, Xing J, et al. Mitochondrial DNA haplogroup M7: a predictor of poor prognosis for colorectal cancer patients in Chinese population. Cancer Sci. 2023;114(3):1056–66. 36382493 10.1111/cas.15654 PMC9986060

[4] Okamoto K, Sasaki K, Nozawa H, Murono K, Emoto S, Yamauchi S, Sugihara K, Ishihara S. Poor prognosis of young male patients with stage III colorectal cancer: a multicenter retrospective study. J Surg Oncol. 2024;129(4):785–92. 38115553 10.1002/jso.27557

[5] Lu Y, Li Z, Zhang S, Zhang T, Liu Y, Zhang L. Cellular mitophagy: mechanism, roles in diseases and small molecule pharmacological regulation. Theranostics. 2023;13(2):736–66. 36632220 10.7150/thno.79876 PMC9830443

[6] Li YY, Qin ZH, Sheng R. The multiple roles of autophagy in neural function and diseases. Neurosci Bull. 2024;40(3):363–82. 37856037 10.1007/s12264-023-01120-y PMC10912456

[7] Wang S, Long H, Hou L, Feng B, Ma Z, Wu Y, Zeng Y, Cai J, Zhang DW, Zhao G. The mitophagy pathway and its implications in human diseases. Signal Transduct Target Therapy. 2023;8(1):304. 10.1038/s41392-023-01503-7 PMC10427715 37582956

[8] Panigrahi DP, Praharaj PP, Bhol CS, Mahapatra KK, Patra S, Behera BP, Mishra SR, Bhutia SK. The emerging, multifaceted role of mitophagy in cancer and cancer therapeutics. Sem Cancer Biol. 2020;66:45–58. 10.1016/j.semcancer.2019.07.015 31351198

[9] Ferro F, Servais S, Besson P, Roger S, Dumas JF, Brisson L. Autophagy and mitophagy in cancer metabolic remodelling. Semin Cell Dev Biol. 2020;98:129–38. 31154012 10.1016/j.semcdb.2019.05.029

[10] Ren Y, Yang P, Li C, Wang WA, Zhang T, Li J, Li H, Dong C, Meng W, Zhou H. Ionizing radiation triggers mitophagy to enhance DNA damage in cancer cells. Cell Death Discov. 2023;9(1):267. 37507394 10.1038/s41420-023-01573-0 PMC10382586

[11] Wang R, Shang Y, Chen B, Xu F, Zhang J, Zhang Z, Zhao X, Wan X, Xu A, Wu L, et al. Protein disulfide isomerase blocks the interaction of LC3II-PHB2 and promotes mTOR signaling to regulate autophagy and radio/chemo-sensitivity. Cell Death Dis. 2022;13(10):851. 36202782 10.1038/s41419-022-05302-w PMC9537141

[12] Goldman MJ, Craft B, Hastie M, Repečka K, McDade F, Kamath A, Banerjee A, Luo Y, Rogers D, Brooks AN, et al. Visualizing and interpreting cancer genomics data via the Xena platform. Nat Biotechnol. 2020;38(6):675–8. 32444850 10.1038/s41587-020-0546-8 PMC7386072

[13] Guo H, Zeng W, Feng L, Yu X, Li P, Zhang K, Zhou Z, Cheng S. Integrated transcriptomic analysis of distance-related field cancerization in rectal cancer patients. Oncotarget. 2017;8(37):61107–17. 28977850 10.18632/oncotarget.17864 PMC5617410

[14] Hu Y, Gaedcke J, Emons G, Beissbarth T, Grade M, Jo P, Yeager M, Chanock SJ, Wolff H, Camps J, et al. Colorectal cancer susceptibility loci as predictive markers of rectal cancer prognosis after surgery. Genes Chromosomes Cancer. 2018;57(3):140–9. 29119627 10.1002/gcc.22512 PMC5778444

[15] Stelzer G, Rosen N, Plaschkes I, Zimmerman S, Twik M, Fishilevich S, Stein TI, Nudel R, Lieder I, Mazor Y, et al. The genecards suite: from gene data mining to disease genome sequence analyses. Curr Protoc Bioinformatics. 2016;54:1.30.31-31.30.33. 10.1002/cpbi.5 27322403

[16] Langfelder P, Horvath S. WGCNA: an R package for weighted correlation network analysis. BMC Bioinformatics. 2008;9:559. 19114008 10.1186/1471-2105-9-559 PMC2631488

[17] Sauerbrei W, Perperoglou A, Schmid M, Abrahamowicz M, Becher H, Binder H, Dunkler D, Harrell FE Jr., Royston P, Heinze G. State of the art in selection of variables and functional forms in multivariable analysis-outstanding issues. Diagn Prognostic Res. 2020;4:3. 10.1186/s41512-020-00074-3 PMC7114804 32266321

[18] Szklarczyk D, Kirsch R, Koutrouli M, Nastou K, Mehryary F, Hachilif R, Gable AL, Fang T, Doncheva NT, Pyysalo S, et al. The STRING database in 2023: protein-protein association networks and functional enrichment analyses for any sequenced genome of interest. Nucleic Acids Res. 2023;51(D1):D638–46. 36370105 10.1093/nar/gkac1000 PMC9825434

[19] Shannon P, Markiel A, Ozier O, Baliga NS, Wang JT, Ramage D, Amin N, Schwikowski B, Ideker T. Cytoscape: a software environment for integrated models of biomolecular interaction networks. Genome Res. 2003;13(11):2498–504. 14597658 10.1101/gr.1239303 PMC403769

[20] Subramanian A, Tamayo P, Mootha VK, Mukherjee S, Ebert BL, Gillette MA, Paulovich A, Pomeroy SL, Golub TR, Lander ES, et al. Gene set enrichment analysis: a knowledge-based approach for interpreting genome-wide expression profiles. Proc Natl Acad Sci USA. 2005;102(43):15545–50. 16199517 10.1073/pnas.0506580102 PMC1239896

[21] Prior FW, Clark K, Commean P, Freymann J, Jaffe C, Kirby J, Moore S, Smith K, Tarbox L, Vendt B, et al. TCIA: An information resource to enable open science. Annu Int Conf IEEE Eng Med Biol Soc. 2013;2013:1282–5. 24109929 10.1109/EMBC.2013.6609742 PMC4257783

[22] Wu C, Zhang Z, Zhang W, Liu X. Mitochondrial dysfunction and mitochondrial therapies in heart failure. Pharmacol Res. 2022;175:106038. 34929300 10.1016/j.phrs.2021.106038

[23] Brown DA, Perry JB, Allen ME, Sabbah HN, Stauffer BL, Shaikh SR, Cleland JG, Colucci WS, Butler J, Voors AA, et al. Expert consensus document: mitochondrial function as a therapeutic target in heart failure. Nat Reviews Cardiol. 2017;14(4):238–50. 10.1038/nrcardio.2016.203 PMC5350035 28004807

[24] Zhao Y, Han J, Hu W, Dai Y, Wu X, Liao X, Zhou H, Nie K. Xiao-Ban-Xia decoction mitigates cisplatin-induced emesis via restoring PINK1/Parkin mediated mitophagy deficiency in a rat pica model. J Ethnopharmacol. 2024;318(Pt A):116882. 37422100 10.1016/j.jep.2023.116882

[25] Yoshida Y, Yasuda S, Fujita T, Hamasaki M, Murakami A, Kawawaki J, Iwai K, Saeki Y, Yoshimori T, Matsuda N, et al. Ubiquitination of exposed glycoproteins by SCF(FBXO27) directs damaged lysosomes for autophagy. Proc Natl Acad Sci USA. 2017;114(32):8574–9. 28743755 10.1073/pnas.1702615114 PMC5559013

[26] Runde AP, Mack R, S JP, Zhang J. The role of TBK1 in cancer pathogenesis and anticancer immunity. J Experimental Clin cancer Research: CR. 2022;41(1):135. 10.1186/s13046-022-02352-y PMC8994244 35395857

[27] Carito V, Bonuccelli G, Martinez-Outschoorn UE, Whitaker-Menezes D, Caroleo MC, Cione E, Howell A, Pestell RG, Lisanti MP, Sotgia F. Metabolic remodeling of the tumor microenvironment: migration stimulating factor (MSF) reprograms myofibroblasts toward lactate production, fueling anabolic tumor growth. Cell Cycle (Georgetown Tex). 2012;11(18):3403–14. 22918248 10.4161/cc.21701 PMC3466551

[28] Sun Y, Shen W, Hu S, Lyu Q, Wang Q, Wei T, Zhu W, Zhang J. METTL3 promotes chemoresistance in small cell lung cancer by inducing mitophagy. J Exp Clin Cancer Res. 2023;42(1):65. 36932427 10.1186/s13046-023-02638-9 PMC10022264

[29] Yao J, Wang J, Xu Y, Guo Q, Sun Y, Liu J, Li S, Guo Y, Wei L. CDK9 inhibition blocks the initiation of PINK1-PRKN-mediated mitophagy by regulating the SIRT1-FOXO3-BNIP3 axis and enhances the therapeutic effects involving mitochondrial dysfunction in hepatocellular carcinoma. Autophagy. 2022;18(8):1879–97. 34890308 10.1080/15548627.2021.2007027 PMC9450969

[30] Qin C, Wang Y, Zhao B, Li Z, Li T, Yang X, Zhao Y, Wang W. STOML2 restricts mitophagy and increases chemosensitivity in pancreatic cancer through stabilizing PARL-induced PINK1 degradation. Cell Death Dis. 2023;14(3):191. 36906621 10.1038/s41419-023-05711-5 PMC10008575

[31] Meng Y, Qiu L, Zeng X, Hu X, Zhang Y, Wan X, Mao X, Wu J, Xu Y, Xiong Q, et al. Targeting CRL4 suppresses chemoresistant ovarian cancer growth by inducing mitophagy. Signal Transduct Target Ther. 2022;7(1):388. 36481655 10.1038/s41392-022-01253-y PMC9731993

[32] Zhao P, Zhen H, Zhao H, Huang Y, Cao B. Identification of hub genes and potential molecular mechanisms related to radiotherapy sensitivity in rectal cancer based on multiple datasets. J Translational Med. 2023;21(1):176. 10.1186/s12967-023-04029-2 PMC9987056 36879254

[33] Liu Z, Liu Z, Zhou X, Lu Y, Yao Y, Wang W, Lu S, Wang B, Li F, Fu W. A glycolysis-related two-gene risk model that can effectively predict the prognosis of patients with rectal cancer. Hum Genomics. 2022;16(1):5. 35109912 10.1186/s40246-022-00377-0 PMC8812245

[34] Khapchaev AY, Shirinsky VP. Myosin light chain kinase MYLK1: anatomy, interactions, functions, and Regulation. Biochem Biokhimiia. 2016;81(13):1676–97. 10.1134/S000629791613006X 28260490

[35] Jin H, Liu B, Guo X, Qiao X, Jiao W, Yang L, Song X, Wei Y, Jin T. MYLK and CALD1 as molecular targets in bladder cancer. Medicine. 2023;102(47):e36302. 38013282 10.1097/MD.0000000000036302 PMC10681608

[36] Tan X, Chen M. MYLK and MYL9 expression in non-small cell lung cancer identified by bioinformatics analysis of public expression data. Tumour Biology: J Int Soc Oncodevelopmental Biology Med. 2014;35(12):12189–200. 10.1007/s13277-014-2527-3 25179839

[37] Halstead MF, Ajtai K, Penheiter AR, Spencer JD, Zheng Y, Morrison EA, Burghardt TP. An unusual transduction pathway in human tonic smooth muscle myosin. Biophys J. 2007;93(10):3555–66. 17704147 10.1529/biophysj.106.100818 PMC2072059

[38] Alhopuro P, Phichith D, Tuupanen S, Sammalkorpi H, Nybondas M, Saharinen J, Robinson JP, Yang Z, Chen LQ, Orntoft T, et al. Unregulated smooth-muscle myosin in human intestinal neoplasia. Proc Natl Acad Sci USA. 2008;105(14):5513–8. 18391202 10.1073/pnas.0801213105 PMC2291082

[39] Ma Q, Xu Y, Liao H, Cai Y, Xu L, Xiao D, Liu C, Pu W, Zhong X, Guo X. Identification and validation of key genes associated with non-small-cell lung cancer. J Cell Physiol. 2019;234(12):22742–52. 31127628 10.1002/jcp.28839

[40] Nie MJ, Pan XT, Tao HY, Xu MJ, Liu SL, Sun W, Wu J, Zou X. Clinical and prognostic significance of MYH11 in lung cancer. Oncol Lett. 2020;19(6):3899–906. 32382337 10.3892/ol.2020.11478 PMC7202280

[41] Wang J, Xu P, Hao Y, Yu T, Liu L, Song Y, Li Y. Interaction between DNMT3B and MYH11 via hypermethylation regulates gastric cancer progression. BMC Cancer. 2021;21(1):914. 34380460 10.1186/s12885-021-08653-3 PMC8359574

[42] Wang R, Xiao Y, Pan M, Chen Z, Yang P. Integrative analysis of bulk RNA-seq and single-cell RNA-seq unveils the characteristics of the immune microenvironment and prognosis signature in prostate cancer. J Oncol. 2022;2022:6768139. 35909899 10.1155/2022/6768139 PMC9325591

[43] Liu S, Wang W, Zhao Y, Liang K, Huang Y. Identification of potential key genes for Pathogenesis and prognosis in prostate Cancer by Integrated Analysis of Gene expression profiles and the Cancer Genome Atlas. Front Oncol. 2020;10:809. 32547947 10.3389/fonc.2020.00809 PMC7277826

[44] Mulet-Margalef N, Linares J, Badia-Ramentol J, Jimeno M, Sanz Monte C, Manzano Mozo JL, Calon A. Challenges and Therapeutic opportunities in the dMMR/MSI-H colorectal Cancer Landscape. Cancers 2023, 15(4):1022. 36831367 10.3390/cancers15041022 PMC9954007

