# Supplementary figures and images for: Identification of a mitophagy-related gene signature for predicting overall survival and response to immunotherapy in rectal cancer
Source: BMC Cancer. 2025 Jan 6;25:15. doi: 10.1186/s12885-024-13412-1 (PMC11706142; doi:10.1186/s12885-024-13412-1)

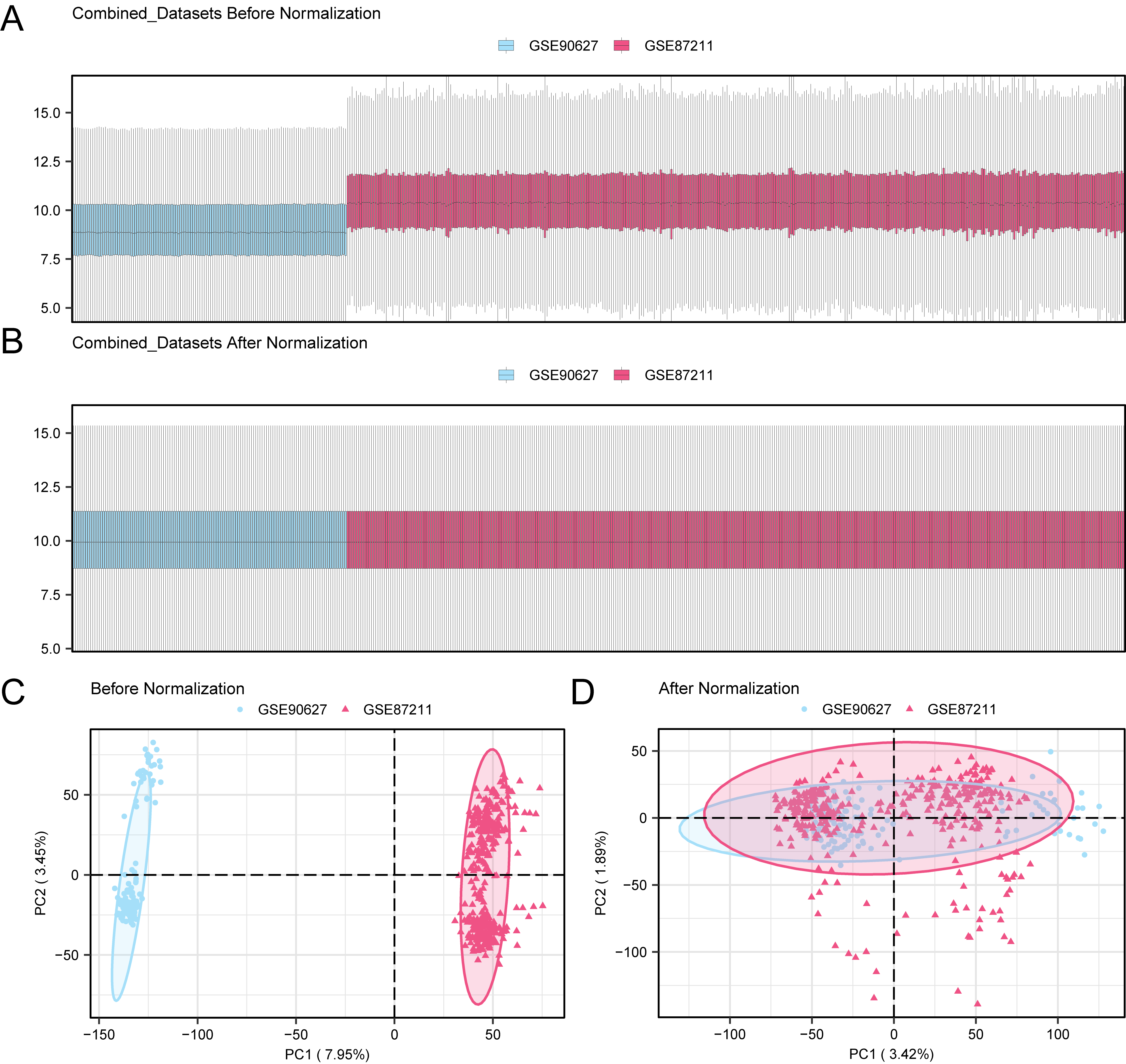

Supplement: Supplementary file 1 — Supplementary Material 1: Supplementary Fig. 1. Removal of Batch Effects from GSE90627 and GSE87211. A. Box plot of the combined GEO dataset distribution before batch removal. B. Postbatch integrated GEO dataset (combined dataset) distribution boxplots. C. PCA plot of the datasets before normalization. D. PCA plot of the dataset after normalization. [file 12885_2024_13412_MOESM1_ESM.tif]

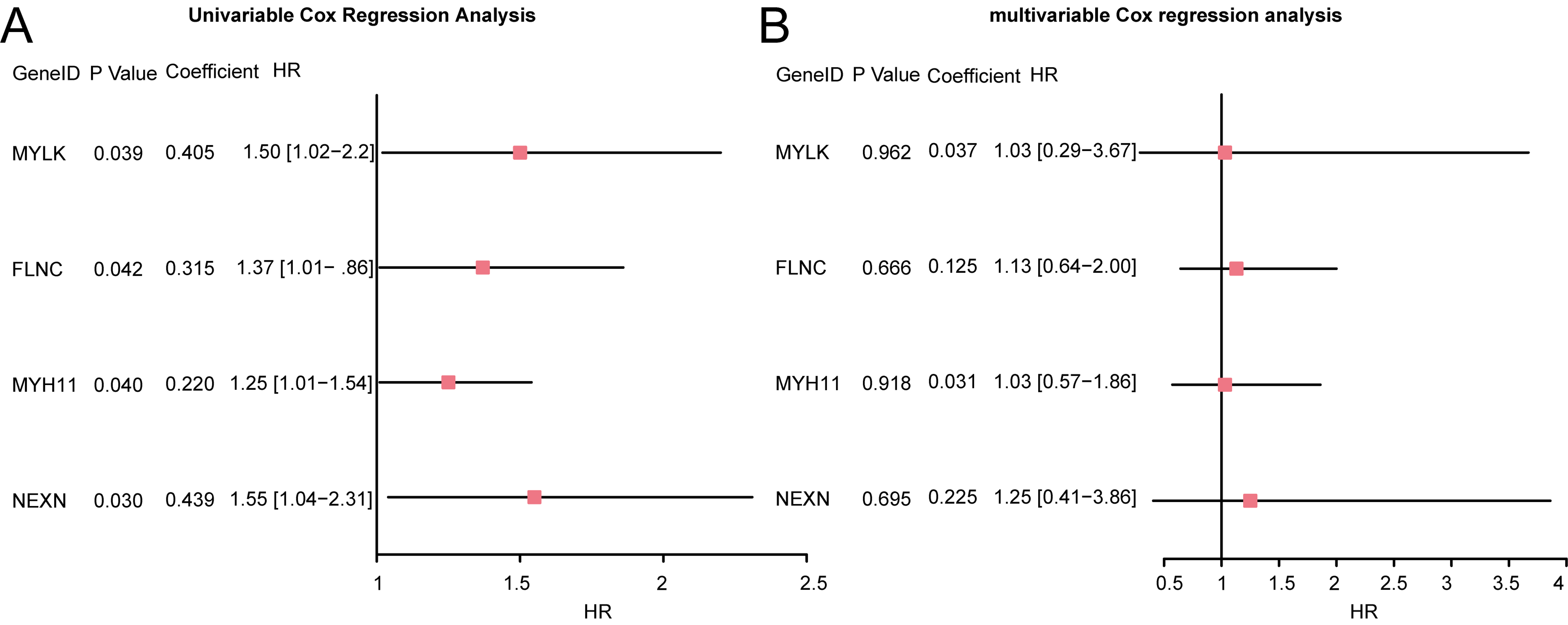

Supplement: Supplementary file 2 — Supplementary Material 2: Supplementary Fig. 2. Cox Regression Analysis. A. Forest plot of the four model genes in the univariate Cox regression model. B. Forest plot of the four model genes in the multivariate Cox regression model. [file 12885_2024_13412_MOESM2_ESM.tif]

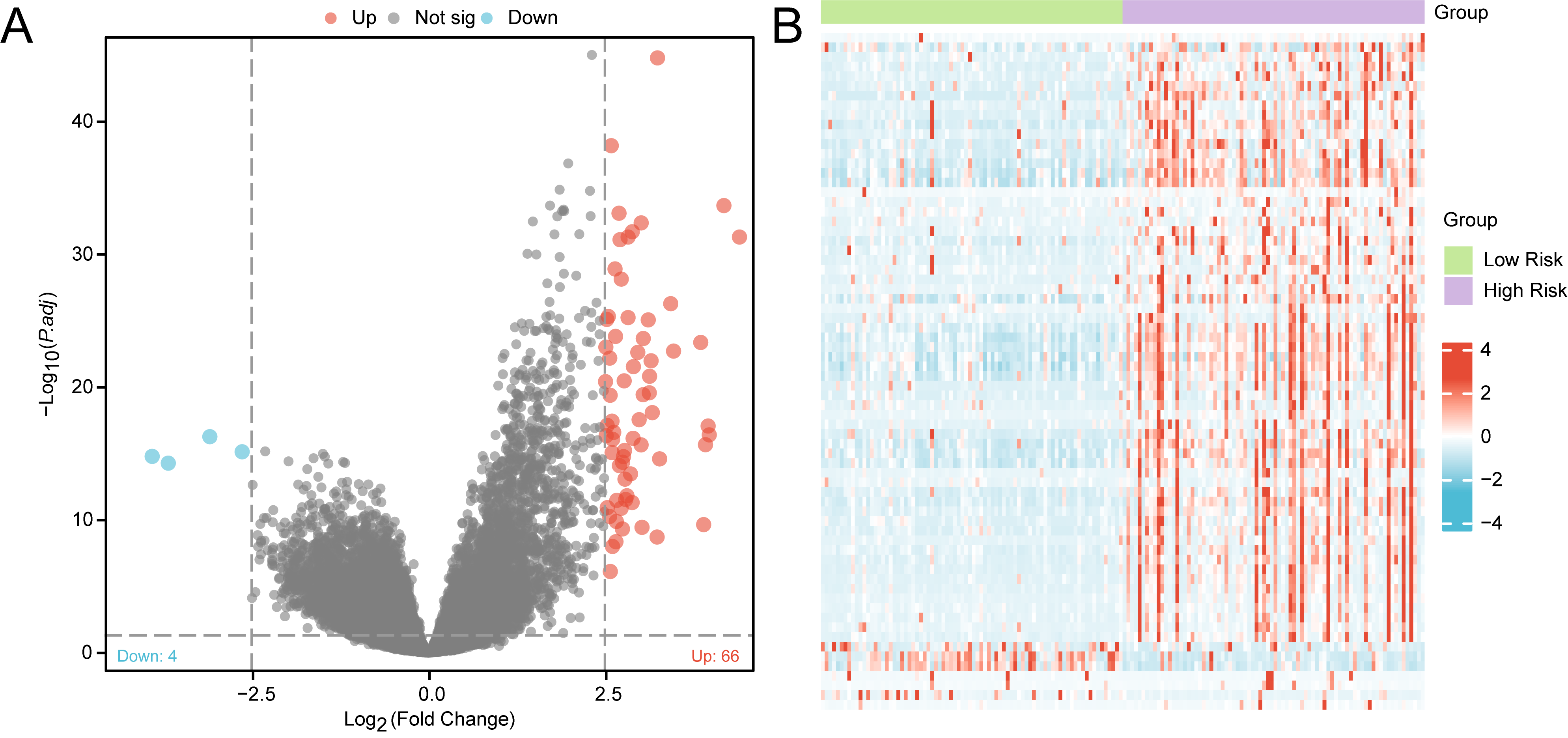

Supplement: Supplementary file 3 — Supplementary Material 3: Supplementary Fig. 3. Differential Gene Expression Analysis for Risk Groups. A. Volcano plot of DEGs associated with high- and low-risk patients in the TCGA-READ cohort. B. Heatmap of DEGs in the high- and low-risk groups. TCGA, The Cancer Genome Atlas; READ, rectal cancer; DEGs, differentially expressed genes. [file 12885_2024_13412_MOESM3_ESM.tif]

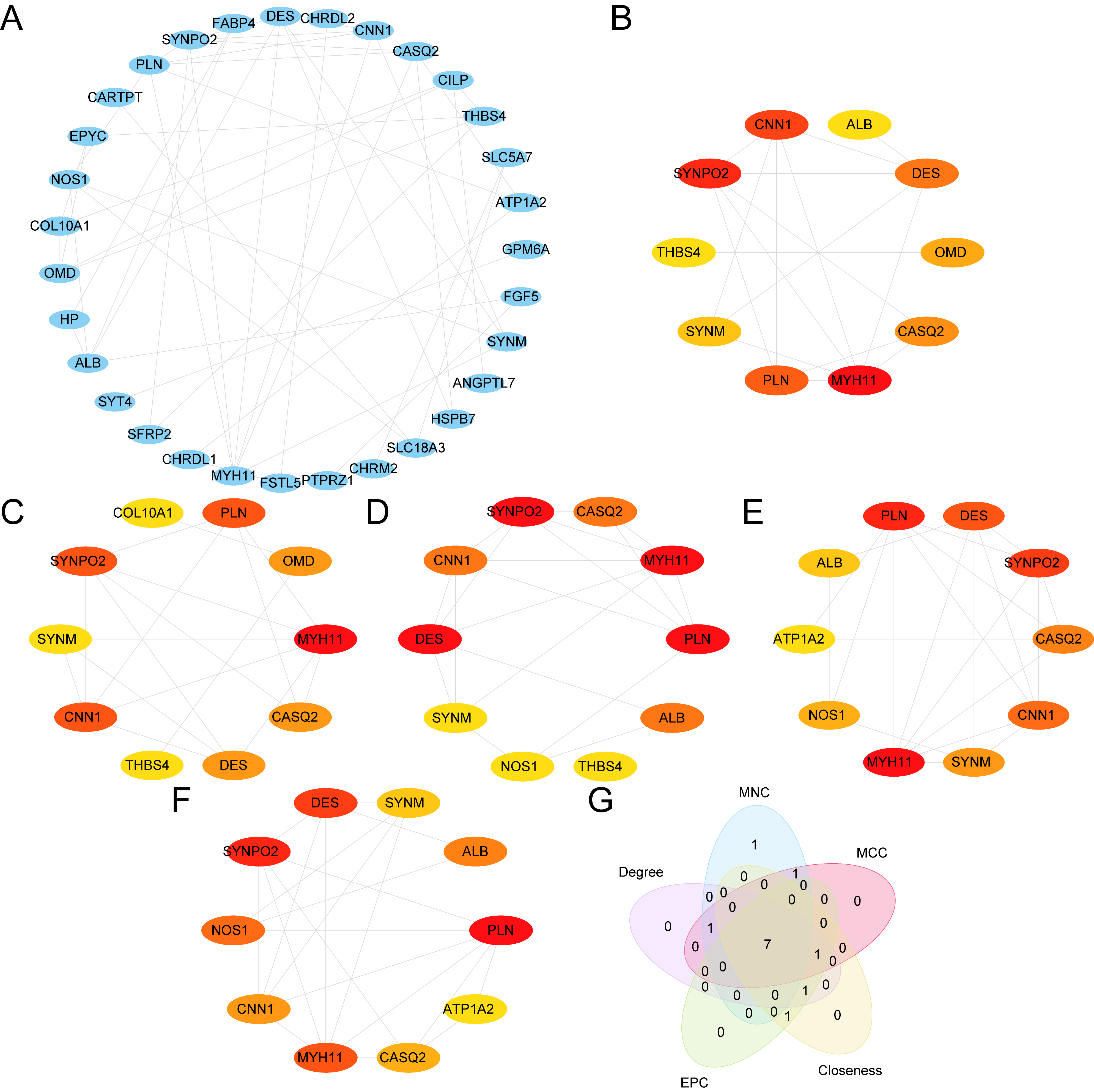

Supplement: Supplementary file 4 — Supplementary Material 4: Supplementary Fig. 4. PPI Network and Hub Genes Analysis. A. PPI network of DEGs in the risk group calculated by the STRING database. B-F. PPI network of the top 10 genes by the 5 algorithms of MCC (B), MNC (C), degree (D), EPC (E) and closeness (F). G. Venn diagram. TCGA, The Cancer Genome Atlas; READ, rectal cancer; PPI network, protein‒protein interaction network; DEGs, differentially expressed genes. [file 12885_2024_13412_MOESM4_ESM.tif]
