# Supplementary material for: Identification of a mitophagy-related gene signature for predicting overall survival and response to immunotherapy in rectal cancer
Source: BMC Cancer. 2025 Jan 6;25:15. doi: 10.1186/s12885-024-13412-1 (PMC11706142; doi:10.1186/s12885-024-13412-1)
Supplement: Supplementary file 5 — Supplementary Material 5. [file 12885_2024_13412_MOESM5_ESM.docx]

**Table 1** **Baseline Table with READ Patients Characteristics**

| **Characteristics** | **overall** |
| --- | --- |
| Age, n (%) | 160 |
| <= 60 | 104 (65%) |
| > 60 | 56 (35%) |
| Gender, n (%) | 160 |
| FEMALE | 71(44%) |
| MALE | 89(56%) |
| Stage, n (%) | 150 |
| Stage I | 30 (20%) |
| Stage II | 48 (32%) |
| Stage III | 48 (32%) |
| Stage IV | 24 (16%) |

READ，Rectal Cancer
