# Supplementary material for: Identification of a mitophagy-related gene signature for predicting overall survival and response to immunotherapy in rectal cancer
Source: BMC Cancer. 2025 Jan 6;25:15. doi: 10.1186/s12885-024-13412-1 (PMC11706142; doi:10.1186/s12885-024-13412-1)
Supplement: Supplementary file 6 — Supplementary Material 6. [file 12885_2024_13412_MOESM6_ESM.docx]

**Table 2 GEO Microarray Chip Information**

|  | **GSE90627** | **GSE87211** |
| --- | --- | --- |
| Platform | GPL17077 | GPL13497 |
| Experiment type | Expression profiling by array | Expression profiling by array |
| Species | Homo sapiens | Homo sapiens |
| Tissue | Rectal Cancer Tissue（READ）；  Normal Tissue（Control） | Rectal Cancer Tissue（READ）；  Normal Tissue（Control） |
| Samples in READ group | 32 | 203 |
| Samples in Control group | 96 | 160 |
| Reference | PMID：28977850 | PMID：38307957 |

GEO，Gene Expression Omnibus；READ，Rectal Cancer
