# Supplementary material for: Identification of a mitophagy-related gene signature for predicting overall survival and response to immunotherapy in rectal cancer
Source: BMC Cancer. 2025 Jan 6;25:15. doi: 10.1186/s12885-024-13412-1 (PMC11706142; doi:10.1186/s12885-024-13412-1)
Supplement: Supplementary file 7 — Supplementary Material 7. [file 12885_2024_13412_MOESM7_ESM.docx]

**Table 3 Results of Cox Analysis**

| Characteristics | Total(N) | Univariate analysis | | Multivariate analysis | | | |
| --- | --- | --- | --- | --- | --- | --- | --- |
|  |  | *P* value | Hazard ratio (95% CI) | | P value | Hazard ratio (95% CI) |  |
| Age | 160 | 0.001 | 1.08 [1.03 − 1.12] | | 0.001 | 1.09 [1.04 − 1.14] |  |
| Stage | 150 | 0.010 | 0.16 [0.03 − 0.86] | |  |  |  |
| Stage I | 30 |  |  | | 0.047 | 0.18 [0.03 − 0.98] |  |
| Stage II | 48 |  |  | | 0.003 | 0.12 [0.03 − 0.49] |  |
| Stage III | 48 |  |  | | 0.014 | 0.17 [0.04 − 0.70] |  |
| Stage IV | 24 |  |  | | 0.571 | 0.70 [0.21 − 2.38] |  |
| Risk.Score | 160 | 0.023 | 2.72 [1.15 − 6.42] | | 0.019 | 3.09 [1.21 − 7.90] |  |
| Gender | 160 | 0.824 | 0.92 [0.42 − 1.99] | |  |  |  |

HR，Hazard ratio
