# Supplementary material for: Identification of a mitophagy-related gene signature for predicting overall survival and response to immunotherapy in rectal cancer
Source: BMC Cancer. 2025 Jan 6;25:15. doi: 10.1186/s12885-024-13412-1 (PMC11706142; doi:10.1186/s12885-024-13412-1)
Supplement: Supplementary file 8 — Supplementary Material 8. [file 12885_2024_13412_MOESM8_ESM.docx]

**Table 4 Results of GSEA for TCGA-READ Risk Group**

| ID | Set Size | Enrichment Score | NES | p value | p.adjust | q value |
| --- | --- | --- | --- | --- | --- | --- |
| REACTOME_EXTRACELLULAR_MATRIX_ORGANIZATION | 291 | 0.708 | 2.892 | 1.00E-10 | 4.75E-09 | 3.37E-09 |
| NABA_CORE_MATRISOME | 255 | 0.705 | 2.857 | 1.00E-10 | 4.75E-09 | 3.37E-09 |
| REACTOME_INTEGRIN_CELL_SURFACE_INTERACTIONS | 83 | 0.786 | 2.782 | 1.00E-10 | 4.75E-09 | 3.37E-09 |
| KEGG_ECM_RECEPTOR_INTERACTION | 83 | 0.779 | 2.754 | 1.00E-10 | 4.75E-09 | 3.37E-09 |
| REACTOME_MUSCLE_CONTRACTION | 186 | 0.680 | 2.665 | 1.00E-10 | 4.75E-09 | 3.37E-09 |
| REACTOME_ECM_PROTEOGLYCANS | 74 | 0.768 | 2.659 | 1.00E-10 | 4.75E-09 | 3.37E-09 |
| REACTOME_COLLAGEN_FORMATION | 90 | 0.733 | 2.610 | 1.00E-10 | 4.75E-09 | 3.37E-09 |
| WP_FOCAL_ADHESION_PI3KAKTMTORSIGNALING_PATHWAY | 288 | 0.574 | 2.343 | 1.00E-10 | 4.75E-09 | 3.37E-09 |
| WP_HIPPOMERLIN_SIGNALING_DYSREGULATION | 111 | 0.552 | 2.022 | 3.00E-07 | 6.34E-06 | 4.50E-06 |
| WP_CLOCKCONTROLLED_AUTOPHAGY_IN_BONE_METABOLISM | 76 | 0.561 | 1.951 | 2.09E-05 | 2.67E-04 | 1.89E-04 |
| REACTOME_CELLULAR_RESPONSE_TO_HYPOXIA | 73 | -0.423 | -1.570 | 3.85E-03 | 2.21E-02 | 1.57E-02 |
| REACTOME_PRE_NOTCH_EXPRESSION_AND_PROCESSING | 96 | -0.438 | -1.693 | 5.32E-04 | 4.32E-03 | 3.07E-03 |
| REACTOME_NEGATIVE_REGULATION_OF_NOTCH4_SIGNALING | 54 | -0.494 | -1.725 | 1.15E-03 | 8.25E-03 | 5.85E-03 |
| REACTOME_SELENOAMINO_ACID_METABOLISM | 117 | -0.615 | -2.450 | 1.00E-10 | 4.75E-09 | 3.37E-09 |
| REACTOME_EUKARYOTIC_TRANSLATION_INITIATION | 119 | -0.620 | -2.482 | 1.00E-10 | 4.75E-09 | 3.37E-09 |
| REACTOME_RESPONSE_OF_EIF2AK4_GCN2_TO_AMINO_ACID_DEFICIENCY | 101 | -0.640 | -2.483 | 1.00E-10 | 4.75E-09 | 3.37E-09 |
| WP_CYTOPLASMIC_RIBOSOMAL_PROTEINS | 88 | -0.657 | -2.492 | 1.00E-10 | 4.75E-09 | 3.37E-09 |
| KEGG_RIBOSOME | 87 | -0.665 | -2.514 | 1.00E-10 | 4.75E-09 | 3.37E-09 |
| REACTOME_EUKARYOTIC_TRANSLATION_ELONGATION | 92 | -0.661 | -2.529 | 1.00E-10 | 4.75E-09 | 3.37E-09 |
| REACTOME_TRANSLATION | 290 | -0.569 | -2.581 | 1.00E-10 | 4.75E-09 | 3.37E-09 |

TCGA，The Cancer Genome Atlas；READ，Rectal Cancer；GSEA，Gene Set Enrichment Analysis。
